# Advancements in Sustainable Livestock Feed: Harnessing Drought-Tolerant Crops

**DOI:** 10.3390/ani16050753

**Published:** 2026-02-28

**Authors:** Sipho Tonisi, Tafadzwa Kaseke, Nqobile A. Masondo, Jerry O. Adeyemi, Olaniyi A. Fawole

**Affiliations:** 1 Postharvest and Agroprocessing Research Centre, Department of Botany and Plant Biotechnology, University of Johannesburg, Auckland Park, P.O. Box 524, Johannesburg 2006, South Africa; cpo.tonisi@gmail.com (S.T.); jerryadeyemi1st@gmail.com (J.O.A.); 2 South African Research Chairs Initiative in Sustainable Preservation and Agroprocessing Research, Faculty of Science, University of Johannesburg, Auckland Park, P.O. Box 524, Johannesburg 2006, South Africa; 3 Agricultural Research Council-Vegetables, Industrial and Medicinal Plants, Roodeplaat, Pretoria 0001, South Africa; masondon@arc.agric.za

**Keywords:** climate-smart agriculture, climate change, alternative livestock feed, drought-tolerant crops, *Opuntia ficus-indica*

## Abstract

Livestock feed shortage is a serious global problem, worsened by climate-change-induced droughts that continue to disrupt its production, consequently threatening food and nutrition security. Drought poses a significant threat to conventionally farmed feed crops, such as maize and soybeans, reducing their availability and negatively impacting the livestock industry. These crops cannot withstand intense drought, creating a need for alternative feed sources with good nutritional value, positive health benefits and livestock performance, as well as cost-reduction potential for farmers. Research continues to explore drought-tolerant crops such as sorghum (*Sorghum bicolor*), millet (*Pennisetum glaucum* and *Eleusine coracana*), cassava (*Manihot esculenta*), false banana (*Ensete ventricosum*), and cactus pear (*Opuntia ficus-indica*) for use as traditional feed substitutes or in hybrid feedstock production to enhance food security, support farmers, and conserve the environment. Unlike the conventional feed crops, these underutilized crops are tolerant under arid conditions, use less water, and possess higher nutritional value, making them important for climate change adaptation and sustainable agricultural systems. Despite the growing recognition of drought-tolerant crops in livestock feed systems, a comprehensive review discussing the advancements and potential of these types of crops as livestock feed is lacking in the literature. Therefore, this review discusses the critical role of selected key drought-tolerant crops as alternative livestock feed, covering the drivers for their use, utilization and processing studies, quality determinants, associated challenges, and sustainable innovation strategies to inform policy making.

## Simple Summary

Feed shortages in arid and semi-arid regions severely threaten sustainable animal production, as conventional feed crops are highly vulnerable to drought. Current research is increasingly exploring drought-tolerant crops as viable alternative feed sources to ensure supply sustainability, yet a comprehensive review detailing the factors affecting their optimal quality and adoption is lacking. Addressing this gap, this review critically discusses preharvest, postharvest, and processing factors affecting the quality of drought-tolerant animal feed sources, alongside evaluating the current state, limitations, and economic potential of these crops. This review establishes that cultivar selection, optimal harvest time, and processing methods such as drying, ensiling, and grinding are critical to maximizing the nutritional and economic benefits of drought-tolerant crops. A transition to alternative crops, including sorghum, millet, and cacti, is identified as a viable economic strategy provided certain key highlighted challenges are effectively addressed. Finally, the review recommends implementing supportive policy interventions, fostering public–private partnerships, and enhancing research capacity to accelerate the adoption of drought-tolerant feed sources, thereby promoting climate-smart animal production.

## 1. Introduction

Climate change has brought devastating impact on agricultural activities that are central to humanity. Climate variability, such as unusual rainfall patterns and rising temperatures, negatively impacts both food and livestock feed crops, leading to food and nutrition insecurity for both humans and livestock [1]. Such developments have spurred research on alternative and sustainable livestock feed sources, with a focus on underutilized plants. The goal of this approach is to identify options that are simultaneously climate-resilient and nutritious for domesticated livestock. The selection of alternative feeds is intended to supplement existing feed sources during periods of shortage, while still ensuring optimal livestock performance and improved profit margins for farmers. Livestock such as cattle, sheep, pigs, and chickens are economically crucial, providing nutritious food, income, and wealth, significantly improving food and livelihoods of the citizens [2,3]. Therefore, solutions that support sustainable livestock production and alleviate the impact of feed shortages due to climate change are warranted.

The conventional livestock feed sources are not sufficient to mitigate feed shortages as their use and availability are further restricted by factors such as land and water use conflicts, feed cost inflations, and the continuous competition between humans and livestock for food sources [4]. The term “conventional feed” in this review covers a range of feedstuffs that may differ depending on the region and farming system. Common, conventional feed sources include but are not limited to forages (grasses and fodder), grains, oilseeds, soybean and sunflower [5]. Feed deficits, primarily due to the impact of climate change on these conventional feed sources, have resulted in the emergence of alternative sources of livestock feed including a wide range of products produced from drought-resilient crops, insects, tree leaves, agro-industrial fruit and vegetable wastes [6,7]. Among these different feed sources, drought-resilient crops are considered a more sustainable option, due to their availability during dry seasons, superior nutritional content, and suitability for small-scale farming systems [8,9]. Drought resilience describes a crop’s overall ability to withstand, survive, and maintain productivity under low water availability. In contrast, drought tolerance, the primary focus of this review refers to a crop’s capacity to endure severe drought through specific physiological or genetic mechanisms. While drought tolerance is a key component of overall resilience, in this review paper the terms are used in their proper, distinct contexts.

The eco-friendly nature, biomass abundance, and affordability of drought-resilient crops further enhance their appeal, particularly for small- to medium-scale livestock production businesses, especially in arid and semi-arid regions. Despite the highlighted benefits for livestock farming, drought-resilient crops have been largely overlooked and underutilized as sustainable feed sources, limiting their usage during drought [10]. Limited utilization of drought-resilient crops stems from limited available research that focuses on their inclusion in feed rations, which can be an opportunity to provide farmers and feed industries with information that can help increase their options for alternative feed sources. Currently, in order to supplement livestock diets, farmers resort to importing feed, which is often more expensive and leads to unsustainable livestock production, especially those of small-medium status. Thus, addressing this important research gap is crucial, given the increasing prevalence of drought and the need for resilient and cost-effective livestock feed options.

Literature has highlighted the potential of several drought-tolerant crops—sorghum (*Sorghum bicolor* (L.) Moench), pearl and finger millets (*Pennisetum glaucum* (L.) R. Br) and *Eleusine coracana* (L.) Gaertn.), cassava (*Manihot esculenta* Crantz), false banana (*Ensete ventricosum* (Welw.) Cheesman), and cactus pear (*Opuntia ficus-indica* (L.) Mill.)-as sustainable alternatives for feed sources, especially during intense drought [11,12,13,14,15,16]. Findings from the above-mentioned studies demonstrate the need to consider drought-tolerant crops as valuable resources to support food and nutrition, food security and climate-smart adaptation. However, the optimal quality of the feed from these crops warrants careful consideration of processing techniques, pre-harvest factors, and maturity stages. These factors interact to determine the nutritional value, digestibility, and overall suitability of the crops for livestock feed, providing critical information required by livestock feed processors. However, studies analyzing the synergistic effects on the quality of the feedstock are still limited.

This review critically discusses the role of key drought-tolerant crops as alternative livestock feed, highlighting the main drivers such as crop usage, processing and application to improve livestock health. Furthermore, factors influencing their quality, challenges, and strategies to promote crop innovation as climate-smart agriculture strategy are highlighted.

## 2. Main Drivers to the Use of Drought-Resilient Crops as Livestock Feed

The primary drivers for the increased use of drought-resilient crops as alternative livestock feed include the impacts of climate change, particularly the effect of drought on conventional feed sources, and the need for sustainable food production systems. Farmers are faced with reduced crop yields and quality due to extreme drought, leading to livestock feed shortages and increased costs of livestock production. Water scarcity and the rise in temperature have heavily impacted the production of conventional livestock feed sources, resulting in a decline in livestock performance and productivity [3]. Various studies have reported on the negative effects of drought on livestock farming across the globe. For instance, Klinck et al. [17] reported a decline in forage and livestock production among small holder and semi-commercial farmers in South Africa. Severe drought in the semi-arid regions of Ethiopia between 1985 and 2011 resulted in significant livestock deaths due to water and feed shortages [18]. In addition, Puerto Rico faced a severe drought that significantly hindered the growth of fodder and grasses, negatively affecting livestock production and farmers’ livelihoods [19].

**Figure 1:**
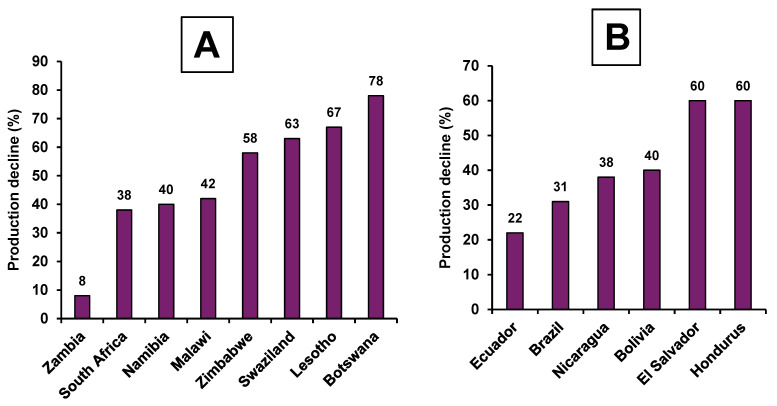
Maize production decline due to the El Niño drought (2015–2016) in (**A**) Southern African, and (**B**) Central and South American countries. Data sources: [20,21,22,23].

Another example of a severe drought occurrence that caused undesired outcomes on livestock production is the 2015–2016 El Niño phenomenon which significantly impacted global crop and livestock productivity, with notable effects in regions like Southern African, South and Central American states [20,21,22,23]. This drought led to a drastic decline (8–78%) in maize production in Southern African nations such as Zambia, South Africa, Zimbabwe and Namibia (Figure 1A). In South and Central American countries like Bolivia, Brazil, Honduras, El Salvador, and Nicaragua, the drought resulted in 31–60% decrease in maize production (Figure 1B). In Southeast Asia, widespread drought-related declines in rice and livestock production triggered a 16% inflation surge in rice-related products and by-products. The 2015–2016 El Niño drought also severely impacted the Philippines, affecting 200,000 maize and rice farmers with an 11% drop in output [24]. Meanwhile, Vietnam experienced losses of over 6000 livestock animals and damage to 70,000 hectares of aquaculture land [19], highlighting the region’s vulnerability to climate extremes. Consequences of the El Niño drought significantly increased the prices of stockfeed, meat and other livestock products, impacting both farmers and consumers. Other related studies reported a 25.8% decline in livestock holdings and 8.4% decrease in milk production in regions such as Ethiopia and a 20% decrease in maize production in Thailand, due to drought-stress-related occurrences [24,25]. Based on past drought experiences, countries such as Vietnam were anticipated to face rice production reduction due to the re-occurrence of the El Niño drought for the year 2023–24 [26]. Also, it has been highlighted that drought can severely decrease nutrient uptake and total protein content in crops such as barley, maize and big bluestem grass [27]. Thus, beyond crop reduction, droughts cause serious disturbances to diets. These factors highlight the far-reaching consequences of drought on food security and livelihoods. Faced with the highlighted challenges, integrating drought-resilient crops into livestock feeding is crucial for sustainable agriculture, especially for livestock productivity. However, understanding the mechanisms behind drought resilience, categorizing and selecting crops based on their drought response is essential for their effective utilization as stockfeed resources.

## 3. Classification of Drought-Resilient Crops

The primary factor in categorizing crop varieties into drought-resilient classes is their ability to withstand different types and intensities of drought, coupled with their mechanism of action that enables them to execute such abilities. This classification aims to facilitate the selection of appropriate crops for sustainable agriculture, ensuring that the chosen varieties can thrive under the conditions under which they can be grown. Although the drought-resilient ability of plants is a multifarious mechanism, the literature has been able to summarize it into four broad categories—(i) drought avoidance, (ii) drought escape, (iii) drought tolerance and (iv) drought recovery. These classifications are derived from varying morphological, physiological and biochemical adjustments plants express to mitigate unwanted impacts caused by drought occurrences [28,29]. The drought-resistance mechanism involves varying degrees of photosynthetic pathways, water-use potential, antioxidant systems, regulation of hormones, leaf and root traits [28]. The previously noted categories are briefly explained as follows:

(i) Drought avoidance: Drought avoidance crops work by keeping water potential elevated at cellular levels enabling them to survive and function even with limited water availability [29]. This is achieved through a combination of drought avoidance and other drought resilience strategies.

(ii) Drought escape: These types of crops are known for rapid flowering which allows the completion of their life cycle before drought conditions become severe [28]. During this period, rapid plant organ development can be achieved due to high rates of metabolic processes that lead to efficient cellular growth and mitosis [30]. In addition to rapid flowering, these plants express early maturity and high photosynthetic rates [31].

(iii) Drought tolerance: These crops can withstand and survive in conditions of low water availability while maintaining the physiological processes necessary for growth and productivity [31,32]. This allows for the cultivation of drought-tolerant crops in arid and semi-arid regions. These plants utilize various mechanisms to cope with water stress, ensuring survival and enabling economic viability.

(iv) Drought recovery: These are the types of crops that resume their growth pathways through the reintroduction of water in their system post a severe drought intensity [33]. This phenomenon includes the ability of the crop to restart nearly all physiological processes that were made dormant by the drought conditions as well as repairing damaged tissues [33].

It must be noted that drought occurrence and plant-response mechanisms are a complex dynamic concept yet to be extensively researched. It has been reported that some plant species are able to execute integrated response mechanisms, signaling a broader adaptability during different drought intensities [28]. As such, the underlying mechanisms respective to each drought-response strategy have been studied. Out of those varying biological processes, drought-tolerant plants with phenomenal photosynthetic pathways have emerged as the next species for sustainable agricultural crop production. Therefore, the following section discusses the types of drought-tolerant crops with efficient photosynthetic pathways and their mechanisms of action against drought.

### 3.1. Types of Drought-Tolerant Crops and Their Mechanisms of Action

Drought-tolerant crops are characterized by effective photosynthetic mechanisms that allow them to thrive in dry and hot conditions. Their ability to concentrate and fix carbon through the conversion of atmospheric inorganic carbon dioxide (CO_2_) into essential organic compounds through specialized carbon-concentrating mechanisms that spatially or temporally separate carbon dioxide (CO_2_) uptake from the Calvin cycle has been implicated in crop resilience against drought stress [28]. Based on carbon fixation and water use efficiency (WUE) pathways, these crops are grouped into three distinct classes: C_3_, C_4_ and crassulacean acid metabolism (CAM) plants [34]. Although these three classes possess similar metabolites, such as malate and pyruvate, their carbon-fixation efficiency varies, influencing their level of drought tolerance. Specifically, CAM plants possess the highest drought tolerance, when compared to C_3_ and C_4_ plant species [34]. Meanwhile, C_3_ plants are generally considered to be less tolerant than C_4_ [35]. Thus, C_3_ plants can be considered less suitable for extremely drought-prone environments. Therefore, this review will concentrate on C_4_ and CAM plants, exploring their unique drought-resilience mechanisms and potential use as alternative livestock feedstock in agricultural systems.

#### 3.1.1. Drought-Tolerant Crops Utilizing the C_4_ Photosynthetic Pathway

**Figure 2:**
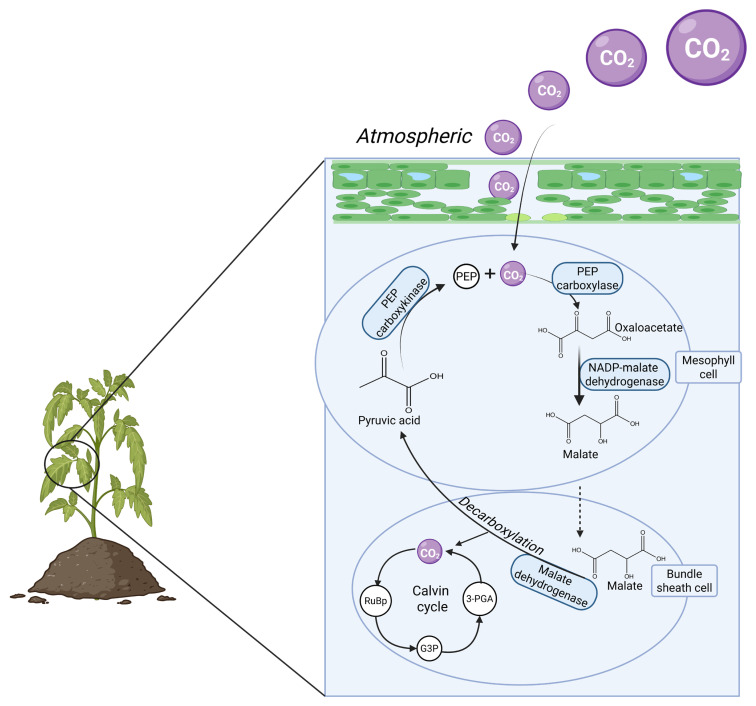
C_4_ photosynthesis pathway of drought-tolerant crops. Abbreviations of metabolites; PEP = phosphoenolpyruvate, NADP = Nicotinamide Adenine Dinucleotide Phosphate, 3-PGA = 3-Phosphoglyceric acid, G3P = Glyceraldehyde 3-phosphate, RuBp = Ribulose 1,5-bisphosphate. Created in BioRender. SPAR (2026) https://BioRender.com/0v9dxll (accessed on 23 February 2026).

The C_4_ plants such as sorghum are highly recommended for livestock farmers in arid and warm climates due to their superior drought resistance [36,37]. These plants convert atmospheric CO_2_ into malate or malic acid—a four-carbon compound—in the mesophyll cell, hence the name C_4_, and transfer it into a bundle sheath cell for decarboxylation (Figure 2). In addition to this exceptional photosynthetic pathway, their ability to thrive in drought-stricken environments has been attributed to reduced interaction and reaction with oxygen molecules, as it leads to unwanted photorespiration, an inefficient process that can result in energy and water loss [38]. Therefore, the significance of C_4_ drought-tolerant plants as alternative feed sources lies in their extended availability during dry seasons, making them one of the key strategies for mitigating feed shortages, particularly among smallholder farmers in arid regions and developing states. Additionally, their efficient water and nitrogen utilization enhances their suitability for pasture production [39]. Compared to C_3_ plants, the C_4_ pathway possesses higher energy-conversion efficiency, which enables them to optimize solar radiation for the production of essential biochemical compounds [39]. This high-energy conversion capacity translates into increased carbohydrate content, which is vital for maintaining livestock metabolism and overall productivity. Additionally, the C_4_ pathway enables these plants to execute photosynthesis even during low CO_2_ concentration, and this is also due to the presence of ribulose-1,5-bisphosphate carboxylase/oxygenase (Rubisco), as illustrated in Figure 2. This allows for efficient use of nitrogen [40], as nitrogen is a vital element needed in plant growth, protein buildup, nucleic acid, amongst many other benefits. Some of the commonly used livestock feed belonging to C_4_ crops include some varieties of maize, and millet [41]. These crops not only serve as primary feed sources but also complement the existing feed formulations in livestock production systems, further enhancing their utility in sustainable livestock nutrition [42].

#### 3.1.2. Drought-Tolerant Crops Utilizing the CAM Photosynthetic Pathway

**Figure 3:**
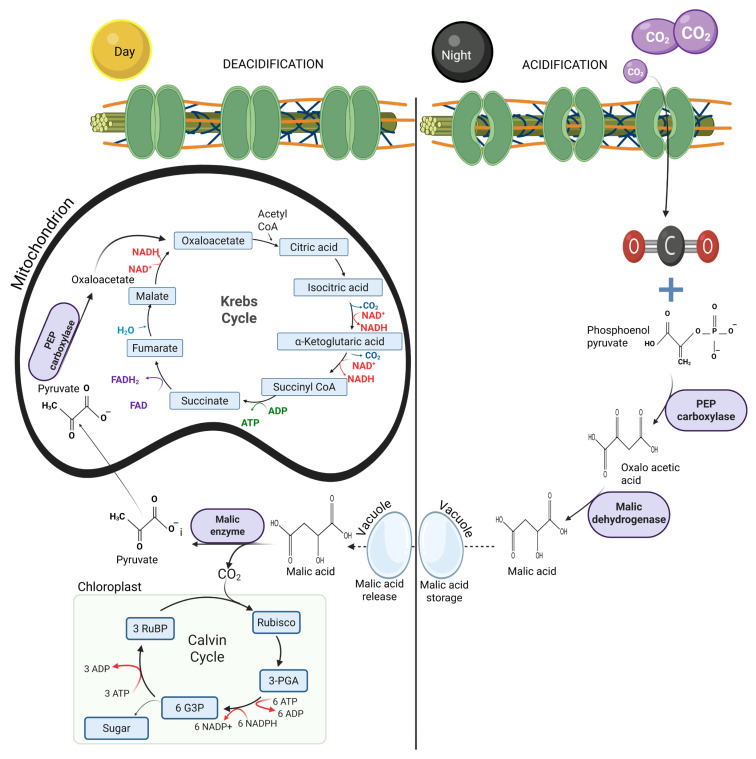
CAM photosynthetic pathway in drought-tolerant crops. Abbreviations of metabolites; PEP = phosphoenolpyruvate, NAD = Nicotinamide Adenine Dinucleotide, NADH = Nicotinamide Adenine Dinucleotide plus Hydrogen, ADP = Adenosine Diphosphate, ATP = Adenosine Triphosphate, FAD = Flavin-Adenine Dinucleotide, FADH_2_ = Dihydroflavine-Adenine Dinucleotide, RuBP = Ribulose-1,5-bisphosphate, NADPH = Nicotinamide Adenine Dinucleotide Phosphate (reduced), NADP^+^ = Nicotinamide adenine dinucleotide phosphate (oxidized), G3P = Glyceraldehyde-3-phosphate, 3-PGA = 3-Phosphoglyceric acid. Created in BioRender. SPAR (2026) https://BioRender.com/8n4roh5 (accessed on 23 February 2026).

Even though CAM and C_4_ plants share similarities in their carbon fixation processes, CAM plants stand out because of their superior water use and conservation properties and their ability to completely prevent wasteful photorespiration [43,44]. Hence, through evolutionary adaptation, CAM plants have developed an efficient survival mechanism that enables them to thrive in extremely arid environments [45]. Their photosynthetic pathway operates through two primary processes: acidification at night and deacidification during the day, as illustrated in Figure 3.

The water- and transportation-use efficiency of CAM plants is further enhanced by their root hydraulic conductivity, which, under wet soil conditions, can be up to six times higher than in other plant species [45]. These types of CAM plants include *Opuntia ficus-indica*, *Ananas comosus* (pineapple), and Agave species which have gained industrial and agricultural significance in recent years [46,47,48]. For instance, the transpiration-use efficiency of *Opuntia ficus-indica* was reported to be 18.57 g/kg, which is 2–6 times higher than those of traditional cereals that have a range of 2–5 and 4–12 g/kg, for plants belonging the C_3_ and C_4_ photosynthetic pathways, respectively [49]. While these crops may have lower protein content than conventional feed sources, they are rich in vitamins, carbohydrates, and essential minerals, making them valuable in producing hybrid feeds [48]. The Food and Agricultural Organization (FAO) associated publication recognizes and promotes CAM plants like *Opuntia ficus-indica* as valuable resources for nutritional properties and recommends their integration into livestock feeding systems as a sustainable alternative feed source during droughts and dry seasons [50,51,52]. This demonstrates how underutilized plants have garnered support across the globe and their potential to revolutionize the livestock feeding systems.

## 4. Recent Studies on the Utilization of Key Drought-Tolerant Crops in Livestock Feeding Systems

Several drought-tolerant crops are crucial in livestock feeding systems, particularly in arid and semi-arid regions. Sorghum (*Sorghum bicolor*), millet (*Pennisetum glaucum* and *Eleusine coracana*), false banana (*Ensete ventricosum*), and cactus pear (*Opuntia* spp.) are notable examples, discussed in this section. Additionally, other drought-tolerant crops such as moringa (*Moringa oleifera*), saltbush (*Atriplex* spp.) and cowpea (*Vigna* unguiculata) are addressed, although existing research on their specific applications remains limited. While drought-tolerant crops are essential for climate-smart agriculture, their nutritional profiles differ significantly from common commercial feed crops; while some nutrients are comparable, others are notably lower, necessitating blended formulations to enhance or maintain nutritional value and functionality (Table 1). Furthermore, processing methods can significantly alter these nutrient levels. Thus, the effect of processing on drought-tolerant crops and impact of the processed product on livestock nutrition and health is also discussed.

### 4.1. Sorghum bicolor (L.) Moench

**Figure 4:**
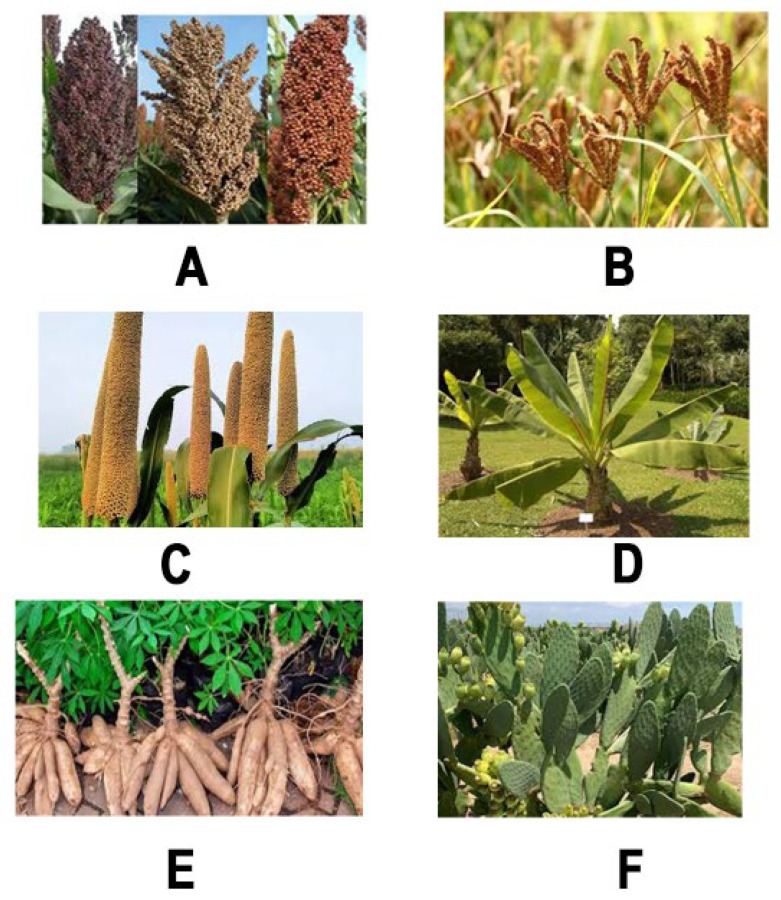
Drought-tolerant crops; (**A**) sorghum (*Sorghum bicolor*), (**B**) finger millet (*Eleusine coracana*), (**C**) pearl millet (*Pennisetum glaucum*), (**D**) false banana (*Ensete ventricosum*), (**E**) Cassava (*Manihot esculenta*) and (**F**) cactus pear (*Opuntia ficus-indica*).

Sorghum (*Sorghum bicolor* (*S. bicolor*)) (Figure 4A) is a prominent drought-tolerant crop, recognized as the most widely cultivated crop for livestock and ranked fifth globally in production, with approximately 65 million tons produced annually [67]. This crop grows well in arid regions of different continents with Nigeria, Zimbabwe, India, China, United States, Mexico, Sudan, Ethiopia, Brazil, Argentina and Australia ranked as the top producers [68]. Besides its drought tolerance, sorghum is further appreciated for its rich nutrient composition, containing 9.5–10.4% crude protein (CP), 1.5% fat, and 6.8% ash, which makes it a better alternative to other feed sources [15,69]. In addition, sorghum is a shorter seasonal crop, and its various parts (leaves, straws, and byproducts) are valuable as supplemental sources for livestock feed rations [69]. As shown in Table 2, various studies have demonstrated the potential of using different parts of sorghum for livestock feed applications. Yusriani et al. [69] reported that using the ensilage technique can assist in extending the postharvest lifespan of sorghum leaves and stems which showed a positive response in livestock feed applications. Within the same study, sorghum grains were seen as a beneficial addition to chicken feed, potentially increasing survival rates at a 30% inclusion level compared to a control group.

Sweet sorghum (12%) produced higher villus heights on the intestinal compartments’ duodenum, ileum and jejunum of male geese [70]. Villus heights of the intestines are key indicators linked to nutrient absorption; as such, the greater the height, the more efficient the nutrient-absorption process becomes, which signifies the quality of the feed produced [71]. Sorghum was also used to replace maize in broiler diets where positive results relating to feed intake, feed conversion and live weight gain during the early days of rearing were observed. The tested inclusion rates were noted to increase weight gains between days 21 to 48 [15]. In addition, the incorporation of sorghum in broiler feed at 30% increased broiler survival rates up to 95.6% compared to the control diet that produced 93.3%. With a similar approach of replacing maize with sorghum, Pontieri et al. [14] demonstrated that sorghum is a viable option for lactating cows and does not compromise livestock performance. High production of compounds such as succinic acid, 2-ethylacrylic acid and glutamic acid were observed during lactation in cows fed with sorghum diets [15,72]. These chemical compounds positively contribute to the quality and flavor of milk products. Besides the nutritional and health benefits, the inclusion of sorghum in livestock is related to its lower costs of production [14]. All these factors put together make sorghum a viable and sustainable crop for livestock feed. Additional studies demonstrating the potential of sorghum as livestock feed are presented in Table 2.

### 4.2. Pennisetum glaucum (L.) R. Br and Eleusine coracana (L.) Gaertn.

Pearl and finger millets (*Pennisetum glaucum* (*P. glaucum*) and *Eleusine coracana* (*E. coracana*)) have been extensively studied as drought-tolerant forages, with various species demonstrating significant utility in livestock feed applications. Their application in the feed industry is attributed to the presence of desirable nutritional properties and bioactive constituents, which renders them ideal for inclusion in feed formulations [73]. For instance, a comparative analysis study by Gowda et al. [74] revealed that finger millet straw contains elevated levels of CP and various minerals, including phosphorus, calcium, copper, and magnesium, better than conventional rice straws. Several millet varieties are documented for livestock feed, with *E. coracana* and *P. glaucum* being the most researched (Figure 4B,C), while millet husks, a byproduct of millet processing, are also less studied [75,76]. These two types are recognized as the predominant groups within the millet category, as they account for a substantial proportion of millet production and trade [73]. Mugula et al. [77] established that *P. glaucum* grains could fully replace maize in the diets of confined cattle, leading to increased dry matter (DM) digestibility and nutrient availability for grazing beef cattle during the dry season. Replacement of maize with various levels of *P. glaucum* resulted in an increased weight gain when fed to broilers [78].

A comparative evaluation between milled and grain diets made from a Gayamba *P. glaucum* showed that whole grains performed better than milled grains, when fed to chickens for 49 days [79]. During the finisher phase, dry feed intake of the treatment that contained 15% whole grain millet increased to 118.77 g compared with 45.18 g recorded during the starter feeding stage. This result is indicative of the adaptability whole-grain pearl millet offers to broiler diets. The increase in dry feed intake was also linked to the increase in daily weight gain as well as feed conversion ratio (FCR). In another study, Oskey [80] demonstrated that Brown Midrib (BMR) pearl millet was preferred over conventional pearl millet when evaluating various nutritional parameters, including neutral detergent fiber (NDF), CP, sugars, and in vitro NDF digestibility. Among these parameters, BMR pearl millet exhibited superior performance in terms of in vitro NDF digestibility, achieving 66.3% compared to 63.6% from conventional pearl millet [80]. These results suggest high fiber in BMR pearl millet which was fermented by rumen gut microbiota into respective health-beneficial byproducts. Using various inclusion levels, millet forms and enzyme addition in pearl millet-based diet, it was observed that a 100% ground pearl millet diet resulted in increased intestinal weight of Japanese quails [81]. This was attributed to increased intestinal morphology due to enhanced functional nutrient absorption and digestion processes in the tested birds. In addition, high contents of fiber can also widen intestinal muscles to allow efficient breakdown of the fibrous components [81]. Other studies demonstrating the beneficial inclusion of pearl millet in livestock treatments with a focus on nutritional value, performance parameters, carcass and histological traits are presented on Table 2.

According to Gowda et al. [74] feeding finger millet straw to dairy cows resulted in a higher average daily milk yield (7.0 L) compared to feeding rice straw (6.3 L), enhanced digestibility of CP, DM, acid detergent fiber (ADF) and NDF. This indicates a potential benefit of finger millet for increasing milk production in cattle and nutrient metabolism. However, a similar study found that incremental replacements of maize with finger millet above 50% in sheep diets decreased the digestibility of DM, organic matter (OM), total digestible nutrients and total carbohydrates relative to using finger millet concentration levels below 50% [82]. This implies that the effect of finger millet may vary depending on the livestock species and its digestive system. Nonetheless, finger millet is still recognized for its high nutritional value, boasting rich protein content and essential minerals like zinc (Zn) [83].

Other studies evaluated various forms of finger-millet-based diets for Mandya lambs, Sahelia goats and cattle [75,76,84]. Performance indices revealed high CP intake of 66 g/day, from the extruded finger-millet-based diet, compared to those made from area sheath and maize cob with values of 56.91 and 60.31 g/day, respectively, when offered to Mandya lambs [76]. Varying proportions of finger millet in silage production proved that the inclusion of 44% finger millet produced reliable results with regard to milk quality and production [75]. According to Tong et al. [84] feeding cattle with millet straw combined with maize showed an increase in fungal population of *Basidiomycota* compared to those fed with maize only. The existence of this rumen fungi in sufficient amounts is associated with an enhanced ability to degrade fiber within the ruminant digestive systems [84].

### 4.3. Ensete ventricosum (Welw.) Cheesman

False banana (*Ensete ventricosum* (*E. ventricosum*)) (Figure 4D), particularly found in Ethiopia, is a drought-tolerant crop used as a feed component for lactating livestock, especially during dry seasons. However, there is a lack of extensive research supporting its widespread use as livestock feed beyond Ethiopia and exploring its full impact on livestock health and farming systems. In spite of this, *E. ventricosum* exhibits characteristics suitable for livestock feed during dry seasons and this is due to its high water content (85–90%), considerable concentrations of 17 amino acids, CP content of 13%, crude fiber (20%), and sugar content of 10%, suggesting that it can be used as fodder or silage [8]. Fiber and stalks obtained from false banana showed considerable amounts of protein and mineral composition (K and Fe), supporting its suitability as a fodder crop [85]. Fekade [86] studied the impact of feeding white leghorn layers and broilers with different rations of *E. ventricosum* replacing maize at varying proportions on DM basis (Table 2). The study revealed that a 30% maize replacement with *E. ventricosum* provided more energy for the chickens leading to increased egg production and profit margins. In another study, various treatments including wheat bran and *E. ventricosum* were offered to Doyogena sheep in a randomized complete design approach and parameters such as feed intake, body weight change, feed conversion efficiency, digestibility and chemical composition were investigated. Notably, treatments that contained higher contents of *E. ventricosum* produced higher contents of metabolizable energy (ME) and in vitro organic matter degradability [16]. Given these promising findings, further studies on the use of *E. ventricosum* in livestock feed are needed to better understand and explore this underutilized drought tolerance resource in various livestock feeding systems.

### 4.4. Manihot esculenta Crantz

Cassava (*Manihot esculenta* (*M. esculenta*) (Figure 4E), known for its drought tolerance and recovery abilities, can be cultivated with minimal inputs in arid regions, making it a valuable crop for food and nutrition security. Its leaves and roots are the main components used for livestock feed, further supported by its ability to regenerate after defoliation caused by prolonged drought conditions. Moreover, the main motivation behind cassava cultivation is that it can be achieved without the inclusion of fertilizers, as outlined by OECD et al. [87]. Cassava can be effectively integrated into livestock feed systems, with various processing and nutritional enhancement techniques such as fermentation. It has been reported that solid-state fermentation of cassava and its residues with *Pleurotus ostreatus* (Jacq.) P. Kumm (oyster mushroom) mycelium increased protein content of the crop from 4.29 mg/100 g to 7.91 mg/100 g after the fermentation process [88]. A cassava foliage that included banana flour and grass hay resulted in increased presence of bioactive compounds in comparison with a foliage that lacked the inclusion of cassava. Amongst the chemical constituents analyzed, chromatographic analysis revealed the presence of isoginkgetin; a major occurring compound known to possess anti-inflammatory potency [89]. Certain novel bacteria, such as *Citrobacter freundii* 5519, isolated from cassava waste have been noted to possess the ability to reduce the occurrence of a toxic cyanide compound, consequently promoting livestock feed safety [90].

The potential of cassava leaves, roots, and byproducts as livestock feed has been studied (Table 2). Ogbuewu et al. [91] found that substituting maize with cassava on the diet fed to chickens significantly improved FCR and average daily gain, with a growth performance of approximately 10%. Evidence from previous studies also showed positive effects of cassava on in vitro gas production, synthesis of volatile fatty acids (VFAs), antipathogenic effect, carcass characteristics and digestibility parameters (Table 2). Evaluation of hematological indices from five-week aged chickens revealed a significant increase in white blood cells (lymphocytes) as a result of including biodegraded cassava root compared to a treatment that contained maize. A 48 h biodegraded cassava root meal recorded lymphocyte content of 47% while the control produced 42% [92]. Replacing up to 50% of feed concentrates with cassava tops and roots in beef cattle diets is a viable option, as it maintains feed intake, nutrient digestibility, and rumen fermentation without negatively impacting average daily gain [93]. Yellow-feather chickens fed 15% cassava-root meal showed similar growth performance (thigh and breast muscle mass) to control birds, indicating that the meal is an ideal partial feed replacement [94]. However, to prevent suboptimal growth performance and carcass parameters, the inclusion levels of cassava in feed diets must be optimized.

### 4.5. Opuntia ficus-indica (L.) Mill.

Cacti (*Opuntia ficus-indica*) species are emerging as a valuable, drought-tolerant livestock feed alternative, offering solutions for livestock health, environmental sustainability, and economic viability, particularly in arid and semi-arid regions. Their high water content, ability to thrive in dry environments, and potential to reduce reliance on traditional, often water-intensive, feed sources make them a promising area of research and application in livestock feeding systems [95]. Among the family of cactus crops (*Cactaceae*), the prickly pear cactus is the most extensively studied species for its potential as a sustainable alternative feed source in livestock systems [47,96,97,98]. Extensive explorations of *O. ficus-indica* (Figure 4F) for various applications, employing diverse plant parts, varieties, and methodologies have been done [97]. As a plant exhibiting remarkable survival and adaptability, different varieties of *O. ficus-indica* have demonstrated the ability to sprout, develop cladodes, and achieve acceptable weight in watershed areas [99]. A proximate analysis of various *O. ficus-indica* varieties revealed CP content ranging from 5.38% to 6.02% [100]. In their assessment of nutritional feed diets, Gebreegziabher and Tsegay [100] observed a notable presence of CP and soluble carbohydrates in *O. ficus-indica*, with values of 83 g/kg DM and 251 g/kg DM, respectively. These figures exceed those of conventional basal diets, which contain 76 g/kg DM and 130 g/kg DM. This disparity proves the value of *O. ficus-indica* as an alternative feed source, given its adequate CP and soluble carbohydrate content, which may be ideal nutrients for gut microbiota, and the energy needs of livestock.

The effect of *O. ficus-indica* as feed on various livestock has been demonstrated producing positive results (Table 2). In goats, incorporating cactus pear silage at a 42% inclusion rate improved ruminating efficiency rate (g DM/h) and water retention while reducing overall water intake without negatively impacting livestock performance [101]. A reduction in water intake is an ideal output when using cactus pear, especially during drought when water supply in farming systems is significantly limited. In the same study, evaluations of livestock productivity indicated that the inclusion of cacti in diets can lead to acceptable weight gains and satisfactory feed intake. Furthermore, the partial substitution of maize grain with *O. ficus-indica* peel powder at inclusion levels of 5%, 10%, and 15% in Cobb chicken diets resulted in improved body weight, feed intake, and FCR compared to the treatment without inclusion of the prickly peel powder [102]. The incorporation of *O. ficus-indica* in livestock feed diets is supported by various studies [12,97,98,103,104]. A recent investigation by da Silva et al. [12] proved that cactus-based diets are able to significantly reduce methane and ammonia nitrogen production (Table 2) better than alfalfa (*Medicago sativa* L.) diets. These results suggest that cactus-based diets are sustainable and environmentally friendly as methane and ammonia nitrogen are considered hazardous to the environment.

Chequer et al. [105] found that adding *O. ficus-indica* mucilage, at varying concentrations, to a lactose egg yolk extender significantly improved boar sperm quality traits such as sperm motility, viability, and membrane integrity. Such observations suggest that the mucilage is a beneficial addition to livestock reproduction. Moreso, adding cactus cladode powder to calf feed at a feed rate of 5 g/day significantly reduced the pathogenic bacteria *Escherichia coli* and *Enterobacteriaceae*, which are known to cause diarrhea in livestock, and such antimicrobial effects were attributed to the high flavonoid content in the cactus cladodes [106]. The formulation of hybrid feed using *O. ficus-indica* combined with other drought-tolerant crops or commercial by-products is recognized as a sustainable and effective strategy to optimize livestock production, particularly in arid and semi-arid regions. While *O. ficus-indica* serves as a vital, water-rich energy source, but its low protein content makes it essential to pair with other feedstuffs for a balanced, sustainable diet. Mixing forage cactus with *Gliricidia sepium* (Jacq.) Walp. (*G. sepium*) hay at inclusion rates of 0–20% improved the nutritional profile of the feed, with maximum concentrations of CP, fats, and DM increasing to 17.19%, 5.66%, and 24.3% at *G. sepium* inclusion levels of 15.38%, 4.94%, and 9.33%, respectively [107]. Furthermore, *G. sepium* hay acts as an effective absorbent, reducing fermentation effluent losses. The forage made from cactus and *Senna obtusifolia* produced high microbial population of lactic acid bacteria (LAB) of 6.3 log CFU/g and lower pH of 4.2, which are indicators of overall good silage quality [108]. When cactus-based forage was combined with cottonseed cake, microbial counts of molds and yeasts remained low, specifically below 2.0 log CFU/g [109]. Precisely, the inclusion of cottonseed cake at 25 and 30% significantly reduced yeast growth to undetectable amounts while increasing aerobic stability, reducing spoilage and nutrient loss. When fed to lambs, this supplementation significantly improved the nutrient digestibility of CP, DM, and OM [109]. For sorghum-based silage, a 90:10 sorghum-to-cactus ratio was recommended to ensure optimal fermentation, digestibility, and chemical composition [110].

### 4.6. Other Drought-Tolerant Crops

Besides the drought-tolerant crops discussed in Section 4.1, Section 4.2, Section 4.3, Section 4.4 and Section 4.5, several others with high nutritional and economic value present significant potential as alternative livestock feed, although their properties are not widely studied. Research has identified Napier grass (*Cenchrus purpureus*), moringa (*Moringa oleifera*), cowpea (*Vigna unguiculata* L. Walp.), and saltbush (*Atriplex* spp.) as valuable, sustainable options for animal-feed applications [111,112,113,114,115,116,117]. Habte et al. [111] demonstrated varying feed-quality performance attributes among 84 Napier grass genotypes under different soil moisture stress levels. While growth declined under severe water stress compared to moderate stress, certain genotypes consistently produced high biomass, improved WUE, increased CP yield, and higher in vitro organic matter digestibility (IVOMD), while maintaining low fiber content (ADF and ADL). Furthermore, ensiling Napier grass with 2% *Lactiplantibacillus plantarum* has been shown to decrease NDF levels, thereby enhancing the nutritional value and increasing the FCR and growth rates in sheep [112]. *Moringa oleifera* has been reported to offer significant positive effects when included in livestock diets, including increased milk yield in dairy cows, improved growth rates in pigs, and enhanced egg production and quality in poultry [113,114]. Furthermore, its ability to act as an antimethanogenic agent makes it a valuable, climate-friendly feed additive for reducing methane emissions in ruminants [113,114]. Despite these benefits, widespread adoption in feed nutrition is limited by high levels of antinutritional factors such as phytates, tannins, and saponins which can reduce nutrient digestibility and lower palatability. While moringa is native to and extensively grown in tropical and subtropical regions like India and parts of China, its potential as a mainstream livestock feed is still constrained by challenges in large-scale cultivation and consistent, sustainable supply chains.

Due to their significant drought tolerance, leguminous crops are being utilized as sustainable, low-cost protein sources in animal feed, particularly for resource-limited farmers. Cowpea, a crop indigenous to Africa, serves as a high-quality alternative to conventional soybeans, offering comparable protein (17–31%) and carbohydrate (50–60%) content, along with essential minerals and amino acids [118,119]. Specifically, in dry-season conditions, the cowpea varieties IN15_62 and IN17_142 have shown superior fodder yield compared to other genotypes [120]. In poultry, diets incorporating 20% raw cowpea have been shown to positively enhance breast muscle fatty acid profiles, particularly by increasing α-linolenic acid, compared to a standard maize-soybean meal diet [121]. Additionally, cowpea inclusion has been associated with a decrease in blood glucose and cholesterol concentrations, prompting recommendations for replacing up to 200 g/kg of soybean meal with cowpea [121]. Similarly, in goats, replacing 50% of conventional feed with cowpea is considered a sustainable option, as it has been linked to higher intakes of CP and ADF compared to other rations [122]. However, like moringa, cowpeas contain antinutritional factors that can reduce protein digestibility [115]. Furthermore, the application of cowpea in animal feed is limited by its dual purpose as a food source for humans, which constrains its availability for large-scale livestock production.

Saltbush (*Atriplex* spp.) is a highly drought-tolerant, halophytic shrub with a long history as animal fodder in the arid and semi-arid regions of Africa, Asia, and Australia [123]. Its primary advantage lies in its ability to grow, and rehabilitate, on soils with high salinity and low water availability. While early reviews by Salem et al. [123] thoroughly documented its nutritional profile, biomass yield, feed application, and inherent limitations (such as high salt and oxalate content), recent data highlighting its evolving role in modern, sustainable livestock systems is lacking. Few recent studies validate its significant value, particularly for camels and small ruminants. Notably, studies indicate that saltbush is exceptionally suited to camels, which can efficiently utilize salty forage. Camels provided with saltbush over 360 days demonstrated improved digestibility of CP and crude fiber, alongside higher nitrogen retention compared to conventional fodder [124]. Furthermore, research suggests that pregnant camels fed saltbush exhibited superior reproductive performance, including increased weight gain, higher milk production, and improved milk quality, specifically characterized by higher lactose and protein content [125]. A recent study indicates that including saltbush in camel diets enhances rumen microbiota, specifically the *Christensenellaceae* R-7 group, which aids in fiber and protein degradation, and *Acetitomaculum*, which helps in neutralizing acidosis [126]. However, despite these benefits, research on saltbush as a primary feed remains limited due to several factors such as extreme levels of minerals resulting in mineral imbalance, high intake of water that can remove feed material in ruminants and accumulation of toxic elements in grazing sheep [123].

Overall, the highlights from Section 4 suggest that current research on drought-tolerant crops primarily focuses on single-crop utilization, with insufficient investigation into nutritional complementarity, palatability changes, and the effects of mixed feeding. A more comprehensive approach is essential to optimize the use of these crops in animal nutrition, particularly when weighing their respective advantages and disadvantages. In addition, studies regarding the application of drought-tolerant crops in animal-feed systems have primarily focused on Africa, the Americas, and Asia. Consequently, there is a significant knowledge gap in other global regions. Diversifying research across different geographic areas is essential to establish robust comparisons and provide a comprehensive global overview of how these resilient plants can be integrated into livestock systems. Furthermore, there is a critical need to transition from subsistence-based cultivation to large-scale commercial farming. Current research indicates that many studies still rely on plants sourced from the wild or small-scale subsistence plots. To truly harness the potential of drought-tolerant crops, priority must be shifted toward commercial-level production, ensuring a stable and scalable supply chain for the animal feed industry.

**Table 2:** Major selected drought-tolerant crops and their application in livestock feed.

Crop	Processing Method	Experimental Model	Targeted Animal	Parameters Measured	Key Findings	Reference
Sorghum	Dried SDDGS	Hereford steers (331.2 ± 33.5 kg) were fed inclusion levels SDDGS (0, 150, 300, 450 g/kg) for 12 weeks against a control (sorghum grain, SFM and urea)	Cattle	Performance indices such as intake and digestibility indices were evaluated.	Inclusion of SDDGS increased intake of CP and GE.	[127]
	BMR mutant lines of sorghum samples were prepared into forage and dried at 65 °C.	In vitro digestion using rumen fluid obtained from two fistulated Friesian Holstein cattle weighing approx. 525 kg.	Cattle	In vitro DM digestibility and in vitro true digestibility were analyzed from the obtained samples.	Cultivar GH2.3 had the highest digestibility of DM. Bioguma and GH2.3 had highest DMI and digestibility. In vitro true digestibility of Bioguma was observed higher than other cultivars.	[128]
	Whole green SW was harvested, chopped and sun-dried.	A total of 144 male Yuzhou goslings (28 days old) were fed with 0, 4, 8 and 12% SW, replacing maize meal.	Goose	Body weight, daily feed intake, ADG, blood composition, antioxidant capacity and intestinal morphology were evaluated.	Increase in SW levels increased ADI and F:G ratio during 28–70 days of feeding.	[70]
	The mixed fodder, with maize as the main component, was prepared by grinding, mixing and pelletizing	ROSS-308 chickens were fed mixed fodder containing 20–40% sorghum grain for 28 days.	Chicken	Carcass characteristics, such as daily weight gain, were monitored during the experiments. Effects on chicken productivity were also observed.	During the second week of chicken rearing, average daily weight gain increased from the diet that consisted of 30% sorghum grain.	[15]
	Supplied sorghum seeds were stored in a dry environment and ground using a roller mill.	Twenty lactating buffalo cows were fed diets with sorghum or maize once a day for about 7 weeks.	Cattle	Milk production, digestibility and carcass scores were analyzed.	Higher milk yield was obtained in the diet that had sorghum compared to the maize diet.	[14]
	Sorghum silage feed diet was developed using an ensiling technique.	Silage of varying proportions from maize and sorghum were fed to mid lactating Holstein dairy cows.	Cattle	Analysis of resultant serum metabolites was conducted on the samples at day 45.	Chromatographic analysis of blood serum revealed presence of linoleic acid, succinic acid, 2-ethylacrylic acid, and glutamic acid levels.	[72]
Millet	Pearl millet samples were ground into powder.	A total of 300 laying hens were fed on a diet that contained various levels of pearl millet replacing 25, 50 and 75% of maize.	Chicken	Every 4th week hen weight and total feed consumption was recorded. Broiler performance was also recorded on starter, grower and finisher broiler.	Broilers that consumed 0–14% whole pearl millet diet had higher body weight gain than those that consumed 28 and 43%.	[78]
	Gayamba pearl millet was purchased from local markets, boiled for 30 min sun dried for 4 days.	A total of Cobb 500 broiler chickens were fed treatments containing starter and finisher diets replacing red sorghum.	Chicken	Daily feed intake, daily weight gain and FCR were measured.	The total feed intake for Gayamba pearl millet diet was observed higher at level 4 during the finisher period.	[79]
	The finger millet sample passed through roasting at 115 °C for 15 min in an oven and ground into powder.	The feed formulations were prepared for chickens housed in well-ventilated cages.	Chicken	Fat deposition assessments.	Fat deposition was reduced while an increase in lean tissue was observed.	[129]
	The straw used was obtained as the result of finger millet processing by product.	The formulated diets had various amounts of finger millet straw which were fed to crossbred rams.	Sheep	Carcass traits, the presence of blood metabolites and the resultant meat quality were assessed.	ADG was higher in livestock that were fed 25% of finger millet straw. Blood metabolite analysis revealed high presence of total protein in the group fed 50% of finger millet straw.	[130]
	The pearl millet was obtained as a whole grain from a local supplier.	Starter and finisher diets were formulated emphasizing millet grains as energy suppliers and fed to 28-day-old broiler chickens, ad libitum.	Chicken	The morphological changes in the structure of the gizzard were recorded.	Increased weight of the gizzard and thick muscles such as *Musculus crassus cranioventralis* and *Musculus crassus caudodorsalis*	[131]
	Black and red finger millet grains were purchased from a nearby market and milled into fine powder.	Feed formulations of 0, 25, 50, 75 and 100% made from black and red finger millet to replace maize were fed to 600 chicks who fed for 28 days.	Chicken	Carcass traits and relevant growth performance indices were assessed.	The chickens that were reared red finger millet had higher final weight compared to those that consumed black finger millet.	[132]
	Millet and maize sample preparation included drying (65 °C) and milling into fine powder before diet formulation.	Diets of different millet and maize combinations formulated before being given to cannulated cows.	Cattle	In situ digestibility, assessment of ruminal composition and microbial diversity were investigated.	Fungal population of *Basidiomycota* when cattle were fed a 50:50 mill: maize combination exponentially increased at 12th hour interval of sample analysis.	[84]
	Finger millet straw samples were formulated using the extrusion technique.	A total of 24 Mandya lambs were fed complete feeds that contained extruded finger millet straw.	Sheep	Daily intake, DMI and FCR were assessed.	Rumen in vitro gas production was observed in finger millet straw compared to extruded feeds from areca sheath and maize cob.	[76]
	Pearl millet grains were sterilized in a saline solution and left to sprout at room temperature for 3 days. Thereafter the sprouts were sun-dried.	A sprouted whole grain millet was incorporated at 25, 50, 75 and 100% in starter and finisher diets of broiler chickens.	Chicken	Growth performance and organ development were analyzed.	Cholesterol levels increased with an increase in sprouted whole grain millet inclusion. Liver weight showed linear response to the incremental inclusion of sprouted whole grain millet.	[133]
	Pearl millet was obtained through the extrusion process and developed into varying degrees.	The extruded pearl millet replaced maize at levels of 25, 50, 75 and 100% and fed to broilers.	Chicken	Carcass performance parameters were recorded.	The treatment with 75% pearl millet achieved better live and breast weight gain.	[134]
	Two methods (grinding and pelleting) were used to create a pelleted and mashed pearl millet-based diet.	Levels of mashed and pelleted millet (50, 75 and 100%) were used to replace maize in broiler chicken diets.	Chicken	The influence of the diet on the growth performance parameters was analyzed.	Pelleted diet did not influence the carcass characteristics of the broilers.	[13]
	Feed rations were made from CORM to replace maize at levels of 0–45% by weight; created through milling to a size of 5 mm.	A total of 192 Cobb chickens were used as experimental livestock, feeding them for 42 days.	Chicken	DMI, ADG and egg production were evaluated.	The 45% enset corm feed resulted in a significantly higher DMI compared to other treatments.	[86]
False banana	*Ensete* sample was chopped and dried using shade drying.	A feed made from replacing wheat bran with enset corm was offered to 42 intact male yearling rams.	Sheep	Livestock feed intake, changes in body weight and feed conversion efficiency were analyzed.	Body weight gain, during the 4th feeding week was with the diets that included 45, 90 and 135 g of enset corm.	[16]
Cassava	Cassava residues were obtained as by products from cassava processing operations.	Cassava residues were included at different levels and fed to Holstein cows.	Cattle	In vitro gas production analysis, analysis of microbial crude protein and ammonia were conducted.	Cassava inclusion increased the pH after 48 h in vitro incubation and decreased production of ammonia-N. Cassava treatments (15, 20 and 25%) resulted in high levels of VFAs.	[135]
	A blend of cassava peel and leaf was created at a ratio of 5:1. Fermentation with water and microbiome added to various diets.	The created blend was then reared into 24 pigs for 16 weeks feeding trial.	Pigs	Growth performance, hematological and biochemical parameters were measured.	The blend made from cassava parts did not negatively affect the measured parameters, suggesting a feasible replacement of maize.	[136]
	Cassava top and root were processed into small chips. Then cassava top-root ratio of 40:60 was processed into silos and ensiled for 14 days.	The silage was fed to beef cattle at different inclusion rates replacing feed concentrates.	Cattle	The pH, chemical composition, fermentation, carcass and blood metabolites were measured.	Digestibility of ADF and NDF were observed to be higher at the silage had 100% of cassava top and root chips.	[93]
	Cassava peel was obtained as a byproduct from the cassava tuber processing.	A 7-week feeding trial consisted of rearing four diets to grower pigs.	Pig	Growth indices were analyzed.	Diet with 50% of cassava peel exhibited gains in total and average weight, total and average daily feed intake.	[137]
	A cassava root meal was obtained.	The cassava root meal of 15, 30 and 45% were assigned to 140-day old chickens	Chicken	Nutrient digestibility, growth performance and organ development were assessed.	The study concludes a diet containing 15% of cassava root meal ideal to attain desired growth indices as well as chicken production.	[94]
	Cassava pulp ensiled with or without the inclusion of *Lactobacillus casei* TH14 (LTH14).	The treatments were then assigned to Thai-native beef bulls.	Cattle	Growth performance and digestibility indices were screened.	The cassava pulp ensiled with *Lactobacillus casei* TH14 (LTH14) produced higher CP digestibility and greater presence of VFAs.	[138]
	The sun-drying method was used to process graded cassava peels to replace maize at treatment levels of 18.5–74%.	Sun-dried cassava peel diets were reared to pregnant goats on a weekly basis.	Goat	Growth performance parameters were analyzed.	Treatment with 37% sun dried cassava peel had higher ADG. Also, a cassava dried peel at 74% was commendable for Soloko goats.	[11]
	A silage made by chopping cassava leaves and then sun dried for 4 h and stacked under anaerobic conditions for 21 days.	Experimental diets were derived by including sun dried cassava leaves at levels of 10–30% before being fed to pigs.	Pig	An analysis of growth and reproductive performance on pregnant pigs.	FCR increased with an increase in cassava leaves.	[139]
	Cassava leaves and peels were fermented with *Citrobacter freundii* 5519.	The fermented cassava peels and leaves were reared to Kamang ducks.	Duck	Changes in carcass traits, growth performance and meat quality were recorded.	Significant increases were observed in parameters such as weight gain and feed intake.	[140]
*O. ficus-indica*	Spineless cacti were procured from a nearby farm.	Varying inclusions of cactus at 20 and 40% were fed to 18 Nellore lambs that had initial weights of 20.5 kg.	Sheep	A 5-day nutrient digestibility trial and proximate analysis of the diets was analyzed.	Nutrient digestibility of OM and DM was higher in treatments that had cactus. Water intake was observed to be low in Nellore lambs.	[141]
	The cladodes were harvested, chopped and used on a fresh basis alongside a forage made from a 5-year-old saltbush.	Awassi sheep were fed two ratios of spineless cladodes and saltbush.	Sheep	Nutritional parameters, digestibility, growth performance and the presence of blood metabolites were screened.	Increasing the levels of cactus and saltbush in diets resulted in an increase in the digestibility of CP.	[142]
	A peel from a ripened fruit of cactus pear was sun dried and processed into powder to allow inclusion at various levels.	Commercial Cobb chickens were fed diets that were made up of prickly pear peels and yellow maize grain.	Chicken	Composition, carcass traits and histopathological results by the diets were investigated.	Feed intake and carcass traits (live body weight and FCR) were better with the diets that comprising 5, 10 and 15% cactus peel powder.	[102]
	*O. ficus-indica* cladodes were sun dried for 7 days and dried at 50 °C for 2 days before being crushed into fine powder.	The ground powders were fed into three groups of chickens with diets that contained 5 and 10% *O. ficus-indica* powders.	Chicken	The effects of *O. ficus-indica* cladode powder were analyzed on the growth and carcass characteristics of the reared chicks.	The 5% inclusion of cladode powder had better body weight gain over the 42-day feeding trial, compared to a control. Carcass characteristics of 10% *Opuntia* powder had better weight gain of liver, gizzard and abdominal fat.	[143]
	*O. ficus-indica* cladodes were tested on a fresh basis by including them in a pig diet.	*O. ficus-indica* was reared in gilts that were in their lactation and gestation phases.	Pig	The influence of *O. ficus-indica* on glucose and insulin concentrations, as well as feed intake was assessed.	The inclusion of *O. ficus-indica* positively influenced the levels of insulin in the blood of the gilts. Also, there was lower body weight loss.	[144]
	Cactus cladodes were dried with direct sunlight for 5 days. The dried cladodes were ground into fine powder using a hammer mill.	Diets were formed with varying inclusion levels of cactus powder and reared to dewormed Nguni heifers.	Cattle	Livestock growth performance, carcass traits and cost benefit analysis were investigated.	The 10 and 20% cactus cladode powder diets had better FCR than control or commercial feed. Cactus powder resulted in reduced expenditure on feed ingredients.	[145]
	Cactus cladodes were harvested during the winter season and sliced before being placed in an oven at 55 °C for a period of two weeks.	The dried samples were fed into a rumen fluid that was collected from fistulated steers.	Cattle	Fermentation patterns, water intake and chemical screenings were conducted.	Cactus diet had 6.64 mg/100 mL of ammonia nitrogen while alfalfa high amounts of 25.1 mg/100 mL. Cactus diet produced lower levels of 2.2% of methane compared to alfalfa (3.52%).	[12]
	The mucilage was obtained from the cladode of *O. ficus-indica.*	The mucilage was applied in sperm freezing extenders at various concentrations using sperm obtained from boar pigs.	Pig	Sperm quality attributes—motility, integrity, viability, morphology and motility—were analyzed.	A 6.7% addition of mucilage improved sperm characteristics such as viability and membrane integrity.	[105]
	The cladodes used in this study were sun-dried and oven dried for 14 and 4–5 days, respectively.	Powdered samples were then assigned to calves at 2.5 g/day.	Cattle	Livestock performance and fecal microbial count were analyzed.	The cladode powder reduced fecal pathogenic population of *E. coli* and *Enterobacteriaceae*, and total coliform count.	[106]

Abbreviations: SDDGS = Sorghum dry distillers’ grains with solubles, BMR = Brown Midrib, SW = Sweet Sorghum, SFM = Sunflower Meal, VFAs = Volatile Fatty Acids. Output variables used to measure growth performance: AWG = Average Weight Gain, ADG = Average Daily Gain. Indices used to measure feed intake performance: DMI = Dry Matter Intake, ADI = Average Daily Intake, intake of CP = Crude Protein, intake of GE = Gross energy. Indices used to measure feed efficiency: F: G = Feed: Gain ratio, FCR = Feed Conversion Ratio. Digestibility indices include the digestibility of: ADF = Acid Detergent Fiber, NDF = Neutral Detergent Fiber, OM = Organic Matter, CP = Crude Protein, as well as In vitro true digestibility. Note: for more animal performance indices refer to Table 2. Intensities include graded inclusions, percentage replacements (DM basis), substitutions, g/kg (DM basis), ratios, addition and dose-based supplementation (g/day).

Table 3 shows a comparison of the carbon footprint, WUE, and cost effectiveness of commercial crops and drought-tolerant crops, demonstrating how drought-tolerant crops can be utilized to promote sustainable agricultural practices in arid and semi-arid regions. However, further research is required to fully understand the long-term sustainability and cost–benefit ratio of incorporating these crops into animal feed, which is essential for guiding evidence-based policy decisions. Because current data relies heavily on estimates, further research is essential to provide policymakers with the evidence needed for informed decision-making. Integrating remote sensing, RGB (Red, Green, Blue) datasets, artificial intelligence (AI) and machine learning into this area of research could significantly enhance data reliability, drive innovation, and introduce “smart” technological frameworks to drought-tolerant crops and livestock production systems. Various RGB datasets, remote sensing and deep-learning methods have been developed to assess crop behavior of agricultural commodities such as cowpea, maize, soybean and wheat under various drought stress levels [146,147,148]. A study by Sang et al. [146] used Unmanned Aerial Vehicle-based RGB data and noted a change, influenced by drought stress, in leaf color and leaf chlorophyll content of soybeans using various vegetation indices obtained from RGB images. The use of an RGB dataset as a basis for a convolutional neural network to learn phenotypic changes in the leaves of maize under different drought conditions managed to produce a model with 88.41% accuracy in terms of detecting water stress levels of the plants [147]. A similar study by Shiva and Gandhi [148] employed machine-learning techniques to quantify moisture-stress levels in chickpea crops, and the developed machine-learning model produced nearly 73% classification accuracy. A recent review by Fares et al. [149] provides up-to-date insights into the use of AI in predicting crop behavior under drought-related conditions. Similar strategies can be employed in collecting reliable data about crop yield, biomass and resilience of drought-tolerant crops during the dry season in order to potentially increase their adoption in various livestock systems.

## 5. Factors Affecting the Quality and Functionality of Drought-Tolerant Crops

This section discusses pre-harvest and post-harvest factors that affect the quality and functionality of drought-tolerant crops. It further highlights how pre-harvest factors such as cultivar and maturity stage, and postharvest factors like processing and storage techniques, impact the quality and functionality of the drought-tolerant crops as livestock feed. In addition, the section explores how these factors collectively inform the economic feasibility for small to medium-sized livestock production businesses, especially in developing nations, where the impact of drought is more severe. These factors provide the foundation for optimizing the quality of drought-tolerant crops-based livestock feed.

### 5.1. Preharvest Factors

#### 5.1.1. Cultivar

While the effect of cultivar type on crop production is well-studied in agronomy, there is a noticeable research gap regarding its influence on drought-tolerant crops. Despite this, there is growing interest in developing drought-tolerant cultivars for sustainable livestock production, as cultivar choice can impact nutrition, livestock performance, and overall drought resilience. Miron et al. [165] compared three newly developed sorghum hybrids (Supersile 20, Silobuster, and Brown-Midrib Hybrid BMR-101) against a commercial variety (FS-5) and observed that the latter performed better than the experimental hybrids exhibiting higher yield, and low levels of residual water-soluble carbohydrates (WSC) after ensiling. In another study, a total of eight sorghum cultivars (Jawar-263, F-1017, Jawar 2002, F-114, Hegari, Sandal-Bar, MR-Sorghum-2011, Pak-China-1) were assessed for their fodder yield characteristics, revealing that parameters such as stem diameter, leaf weight per plant, DM yield, and forage yield were higher in the Puk-China and F-114 cultivars. Additionally, higher levels of crude fiber and CP were observed in cultivars F-7017 and Sandal Bar [166]. Reports from Bean et al. [167], Pinho et al. [168], Neto et al. [169] and Sajimin et al. [170] have demonstrated the influence of cultivar on the nutrient composition, yield, digestibility, agronomic traits, and fermentation profiles of sorghum-derived forages. BMR cultivars exhibited the highest NDF digestibility, while cultivar BRS Ponta Negra showed high contents of WSC, producing greater contents of lactic acid compared to other cultivars [168]. Other studies showing the interaction between sorghum varieties, nutritional, chemical and digestibility parameters have been reported by Wahyono et al. [128] and Kapustin et al. [171].

Several cactus species can be used as livestock feed, including *Opuntia lindheimeri* Engelm, *O. ficus-indica* (L.) Mill., *Opuntia stricta* Haw (*O. stricta*), *Opuntia engelmannii* Salm Dyck., *Opuntia ellisiana*, *Opuntia rastrera* Weber, *O. chrysacantha* Berg, *Opuntia amyclae* (*O. amyclae*) and *Nopalea cochenillifera* Salm Dyck (*N. cochenillifera*). According to Dubeux et al. [96]. *N. cochenillifera* Salm Dyck. exhibited superior WSC, DM, and IVOMD. The replacement of Miúda (*N. cochenillifera* Salm Dyck) with a newly engineered genotype known to be Orelha de Elefante Mexicana (*O. stricta* Haw.) managed to linearly increase microbial protein even though other traits such as DM digestibility, CP, total digestible nutrients and OM decreased with an increase in the replacement proportion of the diets [172]. Conversely, the replacement with Orelha de Elefante Mexicana (*O. stricta* Haw) contributed to the maintenance of milk production in Girolando cows with a recorded measure of 12.5 kg/day. Assessment of nutritional variation amongst *Opuntia* varieties species established that higher levels of pectin were relatively found in erect prickly pear (*O. stricta* Haw) than in Gigante (*O. ficus-indica*), IPA-20 (*O. ficus-indica*), F-08 (*O. atropes* Rose) and African Prickly Pear (*Opuntia undulata*) [173]. According to Ramos et al. [174], higher levels of pectin result in high digestibility of DM of the plant material. Pectin influences cell-wall structure and porosity, facilitating microbial degradation in ruminants and improving nutrient availability. Its role in modulating gut health and binding heavy metals further supports its importance in animal nutrition [174].

In a comparative assessment of genotypes within the *O. ficus-indica* species and those of *N. cochenillifera*, Ramos et al. [175] noted that the cultivars Tamazunchale, Negro Michoacan, and California from *N. cochenillifera*, as well as Orelha de Elefante Mexicana and Amarillo, performed better with respect to dry yield, green mass and WUE. The superior performance of these cultivars can be attributed to the distinct genotypic traits, which include the number of cladodes per plant, cladode dimensions (length, width, diameter, and thickness), as well as overall plant height and width [175]. In this sense, it can be postulated that a greater expression of specific genotypic characteristics correlates with improved performance outcomes. After assessing the performance of cladodes from sixteen cultivars of *Opuntia* based on digestibility and proximate parameters, it was observed that the cultivars Aloqa, Garao, and *Opuntia robusta* var. X11 exhibited the highest levels of similarity (65–98%) when multivariate analysis was conducted [176]. In a proximate analysis of *Opuntia* species, Keyih (*O. ficus-indica* spp.) had the highest DM content recorded to be 91.88%. Ash content was highest in *Opuntia* spp. cultivars Lemats and Sihuna, while *O. stricta* var. Mexicana had the lowest ash content. *Opuntia ficus* and *robusta* species showed the lowest OM content. *O. stricta* var. Wild had the highest NDF (67.05%), and *O. stricta* var. Mexicana had the lowest (37.21%). The reported fiber values were above the recommended ones in South African livestock feed (28%), demonstrating the potential of these studied species and cultivars in enhancing livestock nutrition [177].

Alves et al. [178] analyzed seven *Opuntia* cactus cultivars, focusing on cladode characteristics to understand morphological and nutritional variations. The study observed significant differences in traits like cladode number, length, width, area, and thickness across the cultivars, highlighting their diverse nature. In a rainfed region, the Orelha de Elefante, F-08, and Gigante cactus cultivars demonstrated superior photosynthetic performance compared to other tested varieties (Orelha de Elefante Mexicana, V-19, Redonda, F-08, and Orelha de Elefante Africana, Clone IPA-20) after propagation and harvesting [178]. The larger the total photosynthetic area of the cladodes, the more efficient they are in energy production and bioavailability for the feed livestock. Mineral content, in moderate amounts, of a feed source is essential to the overall nutritional value of a feed diet. For instance, phosphorus is known to be one of the necessary elements for skeletal development, cellular signaling and nucleotide build up, when found in adequate concentrations [179]. Alves et al. [178] found that phosphorus distribution in certain cactus pear cultivars (Giganta, Redonda, V-19, and F-08) is significantly influenced by their genetic differences, with these cultivars showing the highest phosphorus concentration at cladode order 4.

The studies discussed above have demonstrated that morphological traits are positively linked to both dry and fresh mass production, suggesting a strong connection between cultivar and livestock feed outcomes. Thus, breeding programs focusing on these variations could help address feed shortages through optimizing the desirable traits. In addition, cultivar and harvest maturity are closely related because the maturity of a crop at harvest is heavily influenced by its specific cultivar. Meanwhile, different cultivars of the same crop can mature at different rates and reach their peak quality at different times, suggesting that they should be harvested at different stages of maturity.

#### 5.1.2. Harvest Maturity

The maturity of a crop is intrinsically linked to its biological processes, nutrient composition, physical development, and overall plant growth [180]. Consequently, harvest maturity is typically employed as an indicator to ascertain the optimal timing for harvesting of some crops intended for human consumption [181]. Numerous studies have explored the relationship between nutritional composition and harvest maturity in conventional crops, yet there has been comparatively less focus on drought-tolerant crops, particularly those intended for livestock feed.

Four drought-tolerant sorghum cultivars (Early Sumac, Leotti, Nes, and Rox) exhibited varying performances when evaluated at distinct harvesting stages: panicle emergence, milky, dough, and physiological maturity [182]. The study identified the physiological maturity stage as the optimal time for harvest due to the high yield and superior fodder quality by sorghum cultivars [182]. This is probably because the physiological maturity stage represents a period of continued plant development post-harvest, which results in an increased yield and fodder quality, for some plants. However, lower values of ADF (53.9%) and NDF (30%) were recorded during the milky to dough maturity stages, which justified the ensilage of sorghum BRS-610 to improve these attributes as these harvesting periods ensure acceptable fermentation and good nutritive value [183]. During the assessment of different maturity stages of sorghum silage and fodder, the hard dough and physiological maturity stages exhibited better nutritive value and fodder yield [184]. More studies demonstrating the influence of harvest maturity stages on sorghum cultivars have been published by Terler et al. [185], da Silva et al. [186], Alatürk [187] and Hakim [188]. Three varieties (categorized according to silage, biomass and grain) commonly utilized for producing whole crop sorghum silage for ruminants were evaluated for their differences in nutritional values (Table 4). It was found that, across all three tested maturity stages, dry matter yield (DMY) was significantly greater in the variety classified under the biomass group compared to the other varieties [184]. Harvesting during the 3-week stage or boot stage resulted in higher CP and total digestible nutrient content, and lower fiber, while the dough stage had more DMY but lower nutritional value [184]. Nonetheless, the study recommended the dough stage as the optimal maturity stage for enhancing nutritional levels in grain sorghum varieties.

Four maturity stages, spaced over three weeks—boot stage, flowering stage, and dough stage—had varying effects on the quality and yield of Johnsongrass. In this study, da Silva et al. [186] observed that the first harvest at three-week and boot stage maturities were characterized by elevated levels of CP concentrations and total digestible nutrients. The study recommended that Johnsongrass should be ensiled before reaching the flowering stage as this is the optimal phase that produces desired silage qualities [186]. An assessment of the quality of sweet sorghum and sorghum-sudangrass hybrid cultivars harvested at early and late growth stages revealed that dry yield increased with plant maturity, recording a remarkable 172.2% increase in forage at the late growth stage [187]. da Silva et al. [186] identified significant agronomic differences between sorghum green fodder and maize harvested on days 6 and 12, with the latter yielding the highest plant biomass, while the former produced the lowest. Furthermore, Moura et al. [189] expanded the investigation into sorghum maturity stages by assessing DM intake, digestibility parameters, and methane production in sheep. Organic and DM digestibility were observed to be induced with an increase in maturity stage that was measured at day 121 of harvest.

Despite some studies reporting that the chemical and physical properties of *O. ficus-indica* are influenced by various maturity stages, they do not provide additional insights regarding its application in livestock feed. This presents an important research gap that needs to be addressed. These studies can serve as a foundational basis for further investigation into the effects of maturity on *Opuntia* spp. in the context of feed utilization. Research conducted by Juhaimi et al. [190] demonstrated that prickly pear fruits harvested at 15-day intervals from June 15 to August 15 exhibited significant variations in their fatty acid composition and bioactive compounds. Notably, the highest concentrations of phenolic compounds, measuring 156.77 ± 0.09 mg GAE/100 g, were recorded from the harvest of July 1, after which a decline to 121.61 ± 0.09 mg GAE/100 g was observed [190]. In contrast, antioxidant activity, as measured by inhibition against 2,2-diphenyl-1-picrylhydrazyl (DPPH), increased with advancing maturity. This discrepancy may be attributed to the role of other bioactive compounds, in addition to phenolics, that might also contribute to antioxidant activity. Furthermore, the fatty-acid composition revealed the emergence of compounds such as linolenic, behenic, and erucic acids from the harvest of 1 July through to 15 August [190].

Cladodes from various *Opuntia* species, cultivated at different developmental stages for use in ruminant feeding, demonstrated that parameters such as DM, NDF, and ADF increased with maturity across all tested varieties, while CP exhibited an inverse relationship [173,191]. The elevated levels of NDF and ADF may indicate reduced digestibility of the feed, attributable to the composition of plant cell-wall structures, specifically cellulose and lignin, which are typically resilient to breakdown by digestive enzymes and gut microbiota, especially in monogastric animals. A high DM content reflects the availability of other nutrients in the forage, which may enhance the overall nutritive value of the feed. Notably, young cladodes from *O. ficus-indica* (IPA-20) and *O. atropes* Rose (F-08) exhibited high mineral matter concentrations, with respective values of 95.5 g/kg DM and 133.5 g/kg DM. The erect prickly pear (*O. stricta* Haw) also demonstrated substantial mineral-matter content, recorded at 120.0 g/kg DM during the intermediate maturity stage [119]. Furthermore, the study conducted by Da Silva [191] confirmed that the *O. stricta* Haw cultivar contained significant phosphorus levels at the mature stage. Mineral-matter analyses by Pessoa et al. [119] and specific phosphorus content assessments by Da Silva et al. [191] revealed that mineral content increased with maturity in African prickly pear cladodes. Maiuolo et al. [192] investigated the influence of three phenological stages of *O. ficus-indica* cladodes on their antioxidant and anti-apoptotic properties. The findings indicated that early or young cladodes exhibited the lowest antioxidant activity compared to other developmental stages, while medium-aged cladodes demonstrated the highest antioxidant levels and these results were corroborated by the low DPPH inhibition values observed at the same developmental stage. Additionally, the maturity of *O. ficus-indica* showed potential in mitigating apoptosis via the pretreatment of cells with extracts from late-aged cladodes, prior to exposure to lipopolysaccharides for 24 h, which resulted in increased cell survival [192]. These results suggest potential implications for *O. ficus-indica* for enhancing livestock health at a cellular level.

**Table 4:** Effect of maturity stage on the nutritional and chemical characteristics of drought-tolerant crops for livestock feed production.

Crop Type	Maturity Stages	Key Findings	Reference
Sorghum	Milky, milky/dough, dough, dough/dent, dent, hard, dry	DM of the silage increased with grain maturity from 199 to 473 g/kg. Increments in pH levels were observed as a function of grain maturity which ensures good quality conservation of the silage.	[183]
	Panicle emergence, milky, dough, physiological, bloom	As each plant matured, characteristics such as DM, plant height, protein content, and RFV also increased. Physiological maturity stage was advised at the suitable time for harvest as qualities of the fodder and high yield attributes were observed.	[182]
	Bloom, soft, hard, physiological,	Highest DM was obtained when the plant was harvested at physiological maturity. Highest IVDMD was observed at bloom stage. Conversely, fodder made from hard dough maturity stage yielded higher nutritive value when looking at elements such as CP, neutral and acid detergent fiber as well as acid detergent lignin.	[184]
	Late milk, dough, full maturity	DMY was enhanced between late milk and dough stages. Silage made from the whole crop produced a significant increase in ME during late milk and dough maturity stages.	[185]
	3 weeks, boot, flower, dough	The flower maturity stage harvest displayed high content of DM. CP was declared low in the 3-week harvest while high in the dough stage.	[186]
	6th, 12th day	The 12th day harvest produced the highest plant biomass compared to 6th day harvest.	[188]
	Mid-early, late	Crude ash content decreased with an increase in crop maturity.	[187]
	Milk, soft mass, hard mass, mature	Digestibility of DM increased with an increase in crop maturity.	[189]
*O. ficus-indica*	Maturity measured at 15-day intervals.	Phenolic content was found to be 156.77 mg/100 g during the first harvest while the second harvest, known to be last stage maturation, produced elevated antioxidant capacities.	[190]
	Young, intermediate, mature	Young and intermediate phases of African and erect prickly pears species exhibited elevated nutritional parameters that were regarded relevant for ruminant health.	[173]
	Early small sized, young cladodes	Medium-sized and aged cladodes exhibited appreciable antioxidant activity while early harvested cladodes had lower antiradical activity when tested using the ORAC test. Cell viability studies revealed minimal toxicity of the differently harvested cladodes with concentration ranging between 0.01 and 0.1 mg/mL.	[192]

Abbreviation: CP = Crude Protein, DM = Dry Matter, DMY = Dry Matter Yield, ME = Metabolizable Energy, IVDMD = In vitro Dry Matter Digestibility, ORAC = Oxygen Radical Absorbance Capacity, RFV = Relative Feed Value.

### 5.2. Processing Factors

The quality of livestock feed from drought-tolerant crops cannot be fully optimized without the consideration of postharvest management and processing techniques [193]. Properly managed postharvest practices, activities and carefully implemented processing techniques are necessary, as they serve as primary determinants of whether the desired qualities of products derived from fresh produce can be retained, enhanced or degraded [194]. As such, this section focuses on how postharvest factors such as storage, and processing factors such as pulverization, drying and ensiling, impact the quality and functionality of the developed livestock feed products.

#### 5.2.1. Drying Techniques

Numerous studies have demonstrated that drying techniques can impact the quality attributes of various food and feed products. In the context of livestock feed production, however, the application of drying methods to drought-tolerant crops appears to be less prevalent, as evidenced by the scarcity of studies addressing research on drying technologies applied in drought-tolerant forage with the objective of improving feed quality. Among the available drying methods, sun-drying is the commonly utilized technique in the processing of livestock feed. The prevalent application of sun drying in most local farming operations can be due to limited accessibility to innovative drying techniques and machinery. In spite of this, sun drying technique has long proven the capacity of producing livestock feed worth of desired qualities in various farming systems due to its convenience and access. The supplementation of a 5-day sun-dried *Atriplex halimus* L. foliage with exogenous enzymes exhibited a superior DM intake by the sheep, compared to fresh forage that had values of 745 and 396 g/day, respectively [195]. Also, the animals further exhibited higher nitrogen intake from the sun-dried feed (12.1 g/day) than the fresh foliage (6.3 g/day). This investigation posits that sun-dried foliage plus enzymes provide greater palatability than its counterparts, fresh feed and sun-dried crops without enzymes.

Ramsumair et al. [171] investigated the effect of five different drying techniques (oven drying at 70 and 60 °C, sun drying, shade drying and freeze drying) on drought-tolerant species including *Trichantera gigantea* (Bonpl.) C. Mart (*T. gigantea*), *Leucaena leucocephala* (Lam.) de Wit (*L. leucocephala*), *Morus alba* L., and *G. sepium*. Their study recommended shade drying over sun drying for the preservation of feed quality, while freeze drying or oven drying were recommended for laboratory analysis of feed based on the differences in quality attributes observed [141]. Different drying methods produce varying results that could also be influenced by the type of crop processed, on parameters such as ash content, NDF, ADF and acid detergent lignin (ADL). For instance, the freeze-drying option led *Morus alba* to record the lowest values of NDF, ADF and ADL, compared to other plant species dried by the other techniques previously mentioned. The freeze-drying technique could not reduce the NDF of *T. gigantea* as it was the highest amongst all other techniques, with shade drying exhibiting the lowest value of this element. Sun drying was observed to lower the ADF on *T. gigantea* species better than other methods. Oven drying at 60 °C produced the lowest ADF values when dehydrating *G. sepium* plants [171]. The study observed that the interaction between the drying technique and the type of plant species studied played a role in the quality of the final product. Even so, these drying techniques reduced the content of fibrous elements in the samples studied, which is a key indicator of efficient digestibility of livestock forage. However, the relationship between drying methods, volatiles, and fiber remains unclear, as the presence of volatiles within plant matrices is complex and influenced by factors beyond fiber content alone, representing an important research gap requiring further investigation.

Proving how a drying technique is a direct link to feed quality, intake and palatability, solar drying of *G. sepium*, *L. leucocephala*, and *Cenchrus purpureus* (Schumach.) Morrone produced better results in comparison with sun drying. It was observed that solar-dried *L. leucocephala* had a higher DMY of 32.83%, which was double that of sun-dried *L. leucocephala* [196]. Palatability monitored within 3 min was observed higher in solar-dried *G. sepium* leaves and pellets, measuring the highest value of 757 g average intake. Having noted increased CP content and other previously mentioned performance indicators, solar drying with a maximum temperature of 55% was suggested as an effective method of processing plant material to achieve desirable feed quality. A study by Suwignyo et al. [197] found that oven-dried commercial alfalfa for poultry feeding had higher levels of amino acids compared to fresh alfalfa. Essential amino acids such as L-histidine, L-isoleucine and L-valine were relatively similar between the two treatments (oven-dried and fresh), suggesting that temperature (55 °C) did not compromise protein concentration. These findings further suggest that a carefully chosen drying process can improve the nutritional composition of forage or feed. However, sufficient evidence to support such findings is lacking, as research on drying technologies such as freezing drying, hot air drying, oven drying, or their combination in livestock feed is limited, especially on drought-tolerant crops.

Nevertheless, these contemporary drying methods are recognized as the most efficient and convenient agro-processing technologies capable of meeting the demands of the feed industry. For instance, freeze-dried wild cactus cladodes have demonstrated excellent dietary fiber values when processed into flour [198], even though the flour was not intended to be used in livestock feed formulation. Furthermore, freeze-dried wild cactus cladodes exhibited higher concentrations of phenolic compounds and flavonoids compared to other drying techniques, such as tunnel, fluidized bed, and spray drying [198]. Recent research by Ferreira et al. [199] indicated that the food dehydration method could recover higher phenolic content of 0.5 mg GAE/mL in prickly pear, compared to 0.16 mg GAE/mL achieved through microwave drying. Oven-dried (50 °C) cladodes from spineless *O. ficus-indica* f. inermis and spiny *O. amyclae* yielded comparable chemical compositions, VFAs, and gas production parameters during ruminal fermentation studies [200]. Although this investigation did not specifically emphasize the effects of drying concerning ruminant fermentation, it can be reasonably inferred that drying cactus cladodes at 50 °C yields acceptable nutritional values. Notably, a consistent increase in gas production was observed during the ruminant fermentation experiments between winter and summer oven-dried spineless cladodes, with the highest recorded value of 136.8 mL/0.5 g OM from the summer samples after a 72 h period.

Aruwa et al. [201] concluded that freeze-dried *O. ficus-indica* pulp and peel extracts (1–5 mg/mL) exhibit superior DPPH antioxidant activities compared to oven-dried extracts. In the same study, the methanolic freeze-dried extract demonstrated a substantial 18.8 mm zone of inhibition against Gram-negative methicillin-resistant *Staphylococcus aureus* (MRSA), a microorganism recognized as detrimental to livestock health. The observed substantial inhibition of MRSA by the freeze-dried peel supports the notion that drying technology enhances essential biological activities of *O. ficus-indica* within the feed industry. Gouws et al. [202] reported superior performance of microwave-drying (800 W) of prickly pears over freeze-dried (−55 °C and −22 mbar) samples in terms of total phenolic content, recording 149 µg GAE and total flavonoid content of 76.6 µg CE, respectively. A recent comparative study of silage versus solar-dried cactus forage revealed that the latter is more beneficial, as solar-dried cactus forage exhibited higher protein content than ensiled forage while preserving excellent nutritional quality [203]. Studies by Pastorrelli et al. [47] and Maniaci et al. [204] elucidate the significance of dried *O. ficus-indica* in ruminant diets and its substantial contribution to daily milk quality, particularly focusing on cladodes. It is important to note that the drying of drought-tolerant crops remains underexplored, despite the common practice of forage drying on farms. The limited research on the application of drying techniques in feed formulation underscores the need for researchers to initiate investigations aimed at improving forage quality through various methods, including freeze drying, oven drying, and solar drying, either individually or in combination.

#### 5.2.2. Grinding and Pulverization

Mechanical processing during the postharvest phase, both before and after storage, significantly impacts the techno-functional properties, chemical composition, and biological integrity of livestock feed. Grinding has been used as the most suitable mechanical technique in feed technology since it converts a coarse feed into a finer particle-sized powder through physical separation of the fibrous matrix that holds the material intact [205]. The influence of grinding feed on drought-tolerant crops has been studied in relation to feed intake, gut health, nutrient digestibility, growth performance, feed palatability and quality by several authors [206,207,208,209,210,211]. Overall, grinding or pulverizing feed produced positive results towards livestock such as poultry and pigs, with precautionary measures taken into account such as final particle size, the type of grinding equipment, desired feed quality, physicochemical properties of the plant material, and the type of livestock targeted. For instance, in pigs, a feed with particle size of 0.5 mm managed to increase feed efficiency by 6% versus a 0.9 mm particle sized feed [206]. On the contrary, sorghum and barley ground feed with a larger particle size range of 0.3–0.9 mm and 0.43–1.10 mm, respectively, managed to reduce stomach ulcers in pigs [212,213]. Also assessing the effect of sorghum size reduction, from 724 to 319 μm, a study by Paulk et al. [214] noted a linear increase in gain: feed ratio in pigs.

In other livestock sectors, such as poultry, ground sorghum performed better than ground maize, producing liveweight of 2323 g compared to 2206 g observed from ground maize feeding [215]. A whole-grain sorghum increased gizzard weight (47 g) to a greater extent than ground sorghum (39.5 g) [215]. An expanded pellet feed of approximately 3 mm was reported to cause an increase in feed particle size reduction through grinding, consequently reducing the proventricular weight of chickens, ultimately preventing the occurrence of a deadly proventricular dilation disease that impairs nutrient absorption [207]. These pig and poultry studies demonstrate that feed particle size and structure can either enhance or impair livestock performance, depending on several integrated factors.

Research is also exploring the use of varying grinding technologies, and their combination that can turn livestock feed ingredients into powders with better functional properties to livestock [211,216,217,218]. Superfine grinding of beet pulp, to an approximate size of 78 μm, improved properties such as color (lightness) and bulk density than high-speed miller grinding which produced sample sizes of 0.18–45 mm [218]. Grinding sunflower husks have resulted in acceptable increments of essential amino acids and reduction in fiber to appreciable values [216]. In addition, using different designs of crushers (open and closed), has shown that changing sieve parameters has a significant impact on the quality of the processed grain [219]. A normally ground diet with low fiber received high feed intake (76.5 g/day) compared to a low-fiber coarse ground that expressed feed intakes of 67.4 g/day. Also, growth rate and live weight per day were better in this dietary treatment during the weaning days of the tested rabbits [217]. These studies solidify the effect of various grinding and pulverizing methods on livestock feed, performance, and products. However, there is a distinct lack of research concerning the effect of particle size of ground drought-tolerant crops in ruminants, highlighting a significant, unexplored area of study.

#### 5.2.3. Ensiling

**Figure 5:**
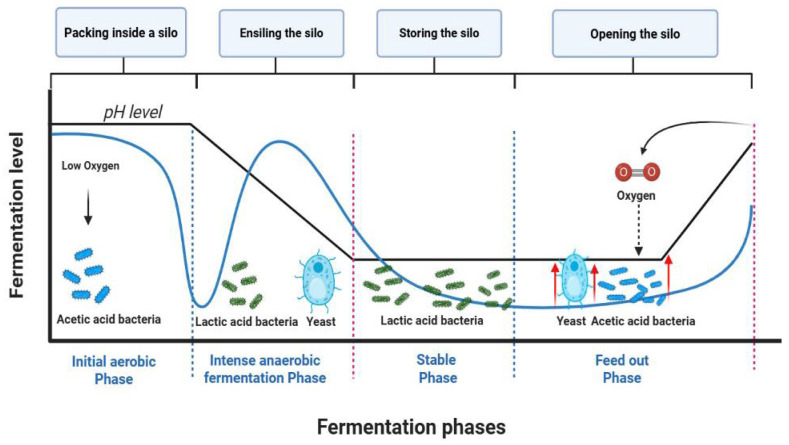
Anaerobic fermentation process occurring under four main phases during the silage production which are initial aerobic phase, intense anaerobic fermentation phase, stable phase and feed out phase. Created in BioRender. SPAR (2026) https://BioRender.com/32wbmkq (accessed on 23 February 2026).

**Figure 6:**
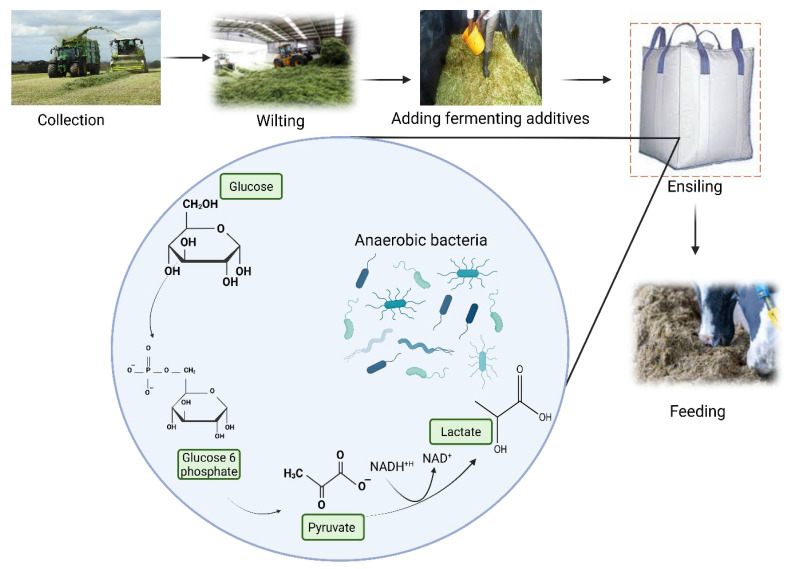
Common silage-making process where a crop or silo is collected and wilted for a period of 24–36 h before the addition of fermenting additives. Created in BioRender. SPAR (2026) https://BioRender.com/bfuumsw (accessed on 23 February 2026).

Silage production, or ensiling is a traditional method for preserving forage quality and ensuring a consistent feed supply, serving as a solution for both drought mitigation and regions prone to heavy, episodic rainfall that makes conventional drying difficult [220]. Due to the adverse edaphoclimatic conditions exacerbated by drought, ensiling of drought-tolerant crops or other forages remains a critical strategy, particularly in agricultural operations that lack adequate processing machinery and storage facilities [221]. The ensiling process is facilitated by natural anaerobic fermentation phenomena largely dominated by LAB and yeasts microorganisms during the four main phases, which are initial aerobic, intense anaerobic fermentation, stable and feed (Figure 5). As demonstrated in Figure 6, these microbes are able to convert OM into various products, predominantly lactic acid, assisted by the absence of oxygen and an acidic pH environment [222]. In addition, ensiling converts unpalatable residues into health-beneficial chemical compounds for livestock, improving rumen degradability of cereal starch and allowing the amalgamation of different feed sources without compromising quality or introducing undesirable complexities [221].

The impact of ensiling on drought-tolerant crops has been studied. Lima et al. [223] demonstrated that ensiling two varieties of sorghum in combination with soybeans, for 30 days at room temperature, yielded superior silage results by improving quality characteristics such as pH and ammonia-nitrogen ratio, compared to silage produced solely from soybeans. The mixed silage produced a more favorable pH outcome (4.03) compared to soybean silage alone (5.47). Lower pH due to higher concentrations of lactic acid is generally preferred for proper silage quality [223]. In addition, a proper silage quality was demonstrated by lower ratios of ammonia nitrogen which were below 4 g/100 g of total nitrogen in the combined silage while the silage of soybean presented a ratio that was above 18 g/100 of total nitrogen. A high ammonia-nitrogen ratio is not a desirable trait when ensiling feed since high ammonia levels indicate potential toxicity of the feed material. These findings confirm that co-ensiling sorghum with soybeans yields favorable fermentation parameters. As silage quality is heavily dependent on the process duration, a 120-day study of sweet sorghum with 30-day interval sampling showed mixed results regarding optimal quality. Although the pH remained consistently below 4, favoring long-term preservation where indicators of efficient fermentation, lactic acid and ethanol gradually increased, and there was a substantial decline in WSC, which dropped from 96.7 to 61.2 g/kg DM [224]. Further increasing the ensiling process to 120 days increased ADF and ADL, which decreased in vitro digestibility of the feed. In a silage prepared from stylo (*Stylosanthes guianensis* (Aubl.) Sw.), a drought-tolerant crop commonly used for livestock nutrition, low levels of ammonia nitrogen and pH after 45 days of ensiling were noted [166]. Optimization of this ensiling technique concluded that the addition of an engineered LAB xg significantly increased in vitro DM digestibility of the forage with values of 68.57% compared to control that had 59.78% [225]. Insignificant degrees of acetic acid are considered an excellent measure of good silage quality. Ensiling varieties of alfalfa have been proven to result in augmented chemical and fermentation qualities of the forage [226]. These studies demonstrate that ensiling duration and addition of supplements results in varying feed features that can be used to optimize nutritional characteristics of livestock feed.

It is worth highlighting that pathogenic bacteria may grow during the ensiling process, and therefore developing additional precautionary measures is advised. In an attempt to address this, Forwood et al. [227] demonstrated that ensiling drought-tolerant sorghum in conjunction with various types of common vegetables such as pumpkin or carrot, at 20 and 40% DM, can allow growth of a beneficial microbial population conducive to livestock health, while suppressing pathogenic microbes. The microbial diversity observed as a result of increased vegetable inclusion was predominantly characterized by *Lactobacillus* species, with a lesser fungal population that included *Kazachstania humilis*, *Monascus purpureus*, *Issatchenkia orientalis*, and *Fusarium denticulatum* [227]. High levels of *Lactobacillus* led to high lactic acid production, which is an efficient compound that can inhibit spoilage-causing microorganisms in livestock feed. Even so, it is of significance to emphasize that the existing literature on the ensiling of drought-tolerant crops underscores the need for further research focused on optimization, integration of diverse feed sources, prevention of microbial spoilage, and the influences of harvest maturity and ensiling duration. These factors may significantly enhance current understanding and practices of ensilage techniques.

#### 5.2.4. Storage

Precise storage conditions after harvest are important for maintaining the quality of agricultural commodities because plant metabolism continues even after the produce is separated from the soil. Proper postharvest handling, including storage, significantly impacts the physiological state and shelf life of the produce [228]. As such, the fundamental aim of any postharvest storage method must be to delay physiological processes that lead to quality deterioration through the limitation of metabolic pathways and pathogenic invasion that favor quality decline, while extending shelf life and maintaining ideal nutritional value [229]. Therefore, the effects of various storage conditions on the quality attributes of drought-tolerant crops require investigations. Temperature is a key factor in postharvest storage, significantly impacting the nutritional value and safety of a feed. High temperatures can accelerate spoilage, reduce nutrient content, and even lead to the growth of harmful microorganisms, affecting livestock health and productivity, while low temperature slows down respiration and metabolic processes [230].

Wang et al. [231] discovered that low temperatures of 5 °C are able to prevent microbial spoilage in wet brewers’ grain by-products from barley that are usually used as livestock feed. Low temperatures have been reported to alleviate the growth and proliferation of *Aspergillus parasiticus*, a microorganism known to cause the generation of aflatoxin in livestock feeds [173]. According to Mannaa et al. [232] temperatures around 8 °C are known to inhibit the occurrence of aflatoxins in grains during storage. During the storage of barley grain feed, high temperatures around 35 °C exhibited the worst surface spoilage after 36 h of storage. Further increase in temperature significantly decreased CP and WSC [231]. The study demonstrated that high storage temperatures can deplete amino-acid content in livestock feed and further cause unwanted growth of fungi, bacteria and insects [233]. A temperature range of 26–37 °C was observed to cause insect infestation, particularly *Rhyzopertha* spp., in sorghum ingredients that are usually used as raw materials for cattle feed [233]. In addition to insect infestation, aflatoxins occur in feed stored under high temperatures posing a threat to livestock health, performance and productivity [234]. Kirigia et al. [235] has drawn conclusive evidence-based perception on how ambient temperature is one of the factors that principally contributes to the decline of quality attributes specific to cowpea. During the analysis it was observed that storing cowpea leaves at an ambient temperature range of 20–25 °C resulted in a decline of starch and sucrose, while there were no noticeable changes observed under 5 °C storage temperature. Alternatively, ambient and high temperatures have been reported as safe for the storage of dry livestock feed sources. Using a closed system, a temperature range of 15–25 °C produced suitable nutritional parameters of lentil grains [236]. Based on the few studies found in the literature, there is a noted research gap regarding the studies on effects of various storage conditions on the final quality of livestock feed made from drought-tolerant crops.

## 6. Challenges of Using Drought-Tolerant Crops as Livestock Feed

Drought-tolerant crops face significant challenges despite their resilience: they remain susceptible to disease infestation, are often characterized by low protein levels and high fiber content and further suffer from low awareness which hinders their widespread adoption as climate-smart agricultural solution. The subsequent section of this review explores these limitations regarding the full integration of drought-tolerant crops into daily farming operations.

### 6.1. Disease and Mycotoxins Infestation, and Feed Safety

Just like any type of agricultural crop, drought-tolerant crops are vulnerable to disease-occurrences. For instance, *O. ficus-indica* cladodes are unfortunately prone to diseases that are difficult to pinpoint to specific pathogens. The high-water content of these cladodes is believed to be a major contributor to disease susceptibility, as it creates a favorable environment for microbial growth [237]. Fungi such as *Alternaria tenuissima*, *Lasiodiplodia theobromae*, and *Fusarium* have been isolated from South African *O. ficus-indica* cladodes, extensively studied and discovered to exhibit symptoms such as chlorosis, necrosis, and black gum exudation [237]. Molecular analyses of fungi isolated from *O. ficus-indica* cladode lesions have confirmed the presence of *Alternaria alternata*, *Colletotrichum gloeosporioides*, *Fusarium lunatum*, and *Curvularia lunata* [238]. Recently, black spot disease has been identified in cactus cladodes cultivated in the Mexican region. Among six fungi responsible for inducing black spot symptoms, three have been classified as significant pathogens: *Alternaria alternata*, *Neoscytalidium dimidiatum*, and *Corynespora cassiicola* [239]. Black spot diseases are typically exacerbated by environmental factors such as high temperatures, conditions that contribute to severe drought intensity. These findings underscore the vulnerability of *O. ficus-indica* to various fungal pathogens, which limit the plant’s yield potential and increase disease transmission to the livestock and possibly humans. During drought conditions, certain diseases may be favored while others are suppressed; however, pathogenic infections remain a significant threat to the diversity of drought-tolerant plants. For instance, charcoal rot disease, caused by the fungus *Macrophomina phaseolina*, has been reported in crops such as sorghum and maize during drought periods [240]. These fungi have developed survival mechanisms that enable them to thrive in extreme heat conditions within dry soil and subsequently migrate towards their target plants. Another fungus, *Aspergillus flavus*, has been implicated in causing premature growth and size reduction in drought-tolerant crops, while fungi from the genera *Fusarium* and *Bipolaris* have been associated with dryland foot and root rot, respectively [240].

In addition to diseases, toxic compounds have also been reported in drought-tolerant crops meant for livestock feed implicated as the major causes of decreased survival rate of these plants. Groundnut shell from *Arachis hypogea* (L.) has been observed to produce aflatoxins through subjecting the seeds to drought-intense conditions [241]. The study concluded that the drought-tolerance ability of groundnut seeds does not help alleviate aflatoxin concentrations, posing a threat to livestock health. Sorghum is also considered to be vulnerable to mycotoxin accumulation as analysis of 1533 samples revealed that a portion of 33% of the crop contained alarming levels of fumonisins, zearalenone, sterigmatocystin, *Alternaria* toxins and aflatoxins [242]. Additionally, factors such as sorghum color, collection period, source and country of origin were seen to significantly contribute to the type, amount and the spread of the analyzed mycotoxins [242]. The rationale behind drought-tolerant crops being susceptible to mycotoxins, especially aflatoxins, can be explained by the climatic conditions of drought, which are extremely hot temperatures and heat stress, that support the growth of mycotoxigenic fungi. Existing edaphoclimatic conditions, characterized by low water availability, reduced nitrogen levels, poor soil quality, and increased toxic metal accumulation, contribute to oxidative stress, phosphate starvation and salt stress, ultimately disrupting the survival, nutrition, and growth of plants [243].

Ultimately, the noted diseases and production of mycotoxins compromise feed safety which lead to limited applications of drought-tolerant crops as alternative feed sources during feed scarcity. The appearance of pathogenic fungi and bacteria in a silage increases the risk of disease transmission to the livestock, consequently affecting the health of livestock and livestock product quality [244]. Institutions such as FAO and International Feed Industry Federation strongly recommend the application of proper storage, preservation and packaging techniques since they hold a valuable position in determining the final quality of the produced feed [245]. Despite ensiling being a common practice to produce feed for livestock, the silage method, however, has been reported as the main host of pathogenic microorganisms, especially when poorly monitored and managed [244]. Frequently occurring disease-causing microorganisms in silage are predominantly *Listeria*, *Salmonella enterobacteria* and *Clostridium* spp. [244]. When silage is subjected to high amounts of moisture, *Clostridium* spp. species develop and convert available carbohydrates into butyric acid instead of lactic acid leading to ketosis that can decrease milk production and weight gain in lactating cows [246]. Thus, when the safety of livestock feed is compromised, it poses health risks not only to the livestock but also to the individuals who consume their products.

### 6.2. Low Crude Protein Content

Protein is a critical nutritional element for livestock feed as it is a pre-requisite for nearly all biological processes in humans and livestock [247,248]. It is a vital feed ingredient for efficient growth performance, tissue repair, milk production, weight gain, and ME, to name a few [249,250,251]. As a result of its implications in livestock productivity, minimum concentration of total protein (measured as CP) in livestock feed has been established. For ruminants, the minimum CP required is reported to be around 7.5%, lower concentrations than that are not advised since they may compromise rumen fermentation [252]. With lactating cows, a diet is recommended to contain about 160 g/kg CP in order to achieve metabolizable protein and reduced nitrogen excretion [253]. In bird husbandry, according to the veterinary manual, CP requirements for broiler rearing ranges from 18 to 23%, depending on the type and age of a bird fed. Using a fitting model, CP requirement was estimated to be around 21% for Jint Tint chicks during a six-week rearing period [55]. For laying hens to achieve desired egg production, CP should be 0.18 g CP/g egg mass [254]. Recently developed CP standards contain contents of 14.7, 22.7 and 35% for pigs, poultry and fish feed evaluation, respectively [255]. These studies cite the importance of a feed source to contain sufficient amounts of CP as part of the recommended dietary attributes, highlighting the point that elevated CP is indeed desirable for livestock nutrition.

However, as reported in Table 5, various studies have shown that a number of drought-tolerant crops meant for livestock feed might fail to meet the minimum requirement for CP content due to the effects of drought and antinutritional compounds, such as tannins, that affect protein synthesis. Nutritional profiles of sorghum varieties (Pant Chari 5, PKV 809 and CSV 17) revealed low CP content of 45.3 g/kg from Pant Chari 5 which also expressed a decline in in vitro dry matter digestibility [256]. In vivo analytical measurements also exhibited low CP intake when sheep were fed the Pant Chari 5 variety. In other studies, it has been revealed that millets are also characterized by low protein content (7.2–7.8%). Pearl millets have been revealed to have low quantities of tryptophan and lysine, which are key amino acids that contribute to protein synthesis [257]. Recent nutritional analysis of millet varieties proved finger millet contains the lowest CP of 7.24%, and similarly, little and kodo millets exhibited respective protein values of 7.6 and 7.8%, making their utilization limited only to ruminants at the minimal CP content highlighted above [202]. Varieties of sweet potato tubers have been proven to contain CP range of 4–6% on DM basis which can be attributed to the occurrence of trypsin inhibitors [258]. *E. ventricosum* tuber revealed CP of 3.33% while the whole plant was reported to contain about 5.98% [8].

These findings suggest the need to blend drought-tolerant crops with conventional livestock feed that contain high CP content. For example, supplementation of finger millet straw with various proportions of noug seed cake resulted in improved body weight gain compared to the control diet that contained finger millet straw only [259], emphasizing the need of protein supplementation in crops such as finger millet. A substitution of rice bran with 9% cassava generated a ruminant meal with approximately 14% CP in DM plus a better protein intake [260]. Likewise, *O. ficus-indica* peel and 12% wheat bran yielded approximately 12% CP in DM [261]. Other approaches include adding fermenting microorganisms to low-CP-containing drought-tolerant crops, are detailed in a recent review by Sukri et al. [48] that the supplementation of *Trichoderma viride*, *Aspergillus niger* and *Saccharomyces cerevisiae* increased CP content in pineapple residues observed from various studies. These studies further demonstrate the importance of feed processing techniques like fermentation and ensiling which can be employed to optimize and enrich the CP in drought-tolerant crops.

**Table 5:** Crude protein content of some of the drought-tolerant crops used in livestock husbandry.

Alternative Feed Source	Crude Protein (% DM)	Targeted Livestock	Reference
Prickly pear silage	3.8	Ruminants	[261]
PT1ecotype *O. ficus-indica* cladodes	6.9	N/A	[262]
PT5ecotype *O. ficus-indica* cladodes	6.8	N/A	[262]
Winter *O. ficus-indica*	4.15	Ruminants	[263]
Summer *O. ficus-indica*	4.19	Ruminants	[263]
Sundried cassava peel meal	3.66	N/A	[264]
Pineapple residue	6	Holstein cows	[265]
Pineapple waste silage	6.2	Myanmar local cattle	[266]
Winter *Agave Americana* L.	5.16	Ruminants	[263]
Summer *Agave Americana* L.	6.30	Ruminants	[263]
Cassava roots	3	N/A	[152]

Abbreviations N/A = Not Applicable. Note: The units are from the studies by Gebremariam et al. [95]; Degu et al. [267] and Rodrigues et al. [262] have been converted to % DM for uniformity as stipulated.

### 6.3. High Lignin Content and Limited Digestibility

**Figure 7:**
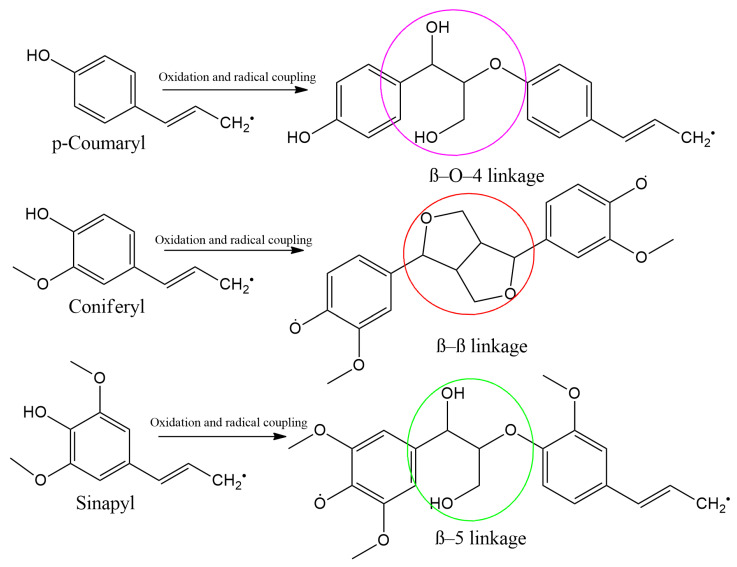
Lignin biosynthesis from phenylpropanoid units (p-coumaryl, coniferyl and sinangyl) resulting in bonds that restrict digestion. Created using Chemdraw (Chemdraw Ultra 12.0).

The survival strategy of drought-tolerant crops, which primarily relies on high lignin composition to conserve water during periods of aridity, adversely affects feed digestibility by enzymes and the gut microbiome in livestock [265]. Although lignin is a necessary cell-wall component whose function is needed during hot temperatures, heat stress, transportation of minerals and mechanical support [266], its presence becomes a physical barrier hindering the microbiome from reaching needed polysaccharides, cellulose, hemicellulose, starch and protein. The chemical make-up of lignin determines its digestibility. Lignin chemical composition includes two major monolignols (Figure 7): dimethoxylated syringyl (S) and monomethoxylated guaiacyl (G). As a result, the ability of these monolignols to form bonds with other units is directly related to a measured indigestibility of plant material in livestock. An increased concentration of G units is believed to enhance plant indigestibility, which may also be associated with greater maturity. It should be noted that lignin and plant digestibility are complex and challenging phenomena as lignin varies across plant species, also the method to measure the influence of lignin on digestibility cannot be easily applied due to various factors such as crop age, environmental conditions and plant morphology, to name a few [265].

The inability of rumen bacteria to efficiently degrade lignified plant cell wall limits the conversion of cellulose and hemicellulose to short fatty acids which play a crucial role in energy provision for livestock, whereby lignin itself contains energy that is 30% higher than that of cellulose [265]. Highly lignified feed formulations extend the passage time of feed within the ruminal digestive system, thereby reducing feed intake. This reduction in feed intake can be correlated to suboptimal livestock performance. The influence of lignin on feed digestibility has been extensively researched over the years, with various studies demonstrating a negative correlation between lignin concentration and digestibility [268,269,270]. For instance, increasing lignin concentration (9.59–13.3%, NDF basis) in feed diets reduced the digestibility of NDF, CP, and starch in lactating cows [271]. Supporting the notion that feed digestibility may also depend on the digestive system of the livestock, an in vivo study involving buffalo demonstrated excellent ADL degradability [272], which can be assumed to be potentially facilitated by microorganisms such as *Rikenellaceae* RC9 gut group, *Ruminococcaceae* UCG-011 and *Prevotella* due to their abundance and associated fiber degradation ability in the rumen [273]. The extent to which the lignin undergoes degradation and affects digestibility may also depend on its structural composition, specifically highlighting which subunit predominates. Feeds with a high presence of the sinapyl unit exhibited enhanced digestibility of ADF, NDF, and cellulose, suggesting that the linearity of sinapyl promotes efficient lignin degradation [272].

High concentrations of lignin and their detrimental effects on feed digestibility of drought-tolerant crops have led to the development of pretreatment techniques aimed at reducing or removing lignin to make cellulose and hemicellulose more accessible for gut microbiota [274,275]. Several authors have reported on methods to lower lignin concentrations specifying pretreatments such as comminution, application of rot fungi, the use of chemicals such as potassium hydroxide, sodium hydroxide, calcium hydroxide, sulphuric acid and physical pretreatments such as soaking and mechanical grinding [276]. Escapa et al. [277] and Jędrzejczyk et al. [278] have further reported on the use of grinding, pyrolysis (using temperature greater than 300 °C), microwave oven, hot water, acid or alkaline pretreatment. Pretreatments created from enzymatic systems are also reported as a viable option able to decrease lignin content in livestock feed [279]. The common objective of these cited pretreatment applications is to disrupt the cross-links present in lignocellulosic compounds to facilitate the availability of essential nutrients, such as sugar polymers, for further efficient digestion in livestock. Although such pretreatments may seem promising, their application for feed purposes may be hampered by various factors, for instance, the fungal treatment is limited by long incubation time (approximately 7 weeks), and variation in biomass and fungal strains that make choosing the best microbe difficult [280]. Chemical methods such as acid pretreatments lead to undesirable inhibitory byproducts that block microbial digestion, which is essential for breaking down carbohydrates [281]. Physical methods, on the other hand, require intensive energy input to operate the grinding equipment with lower economic returns [282]. These disadvantages have contributed to the limited application of these pretreatments.

### 6.4. Socio-Economic and Financial Challenges

The adoption of drought-tolerant crops faces several interconnected challenges. These include insufficient knowledge and training, lack of awareness, limited availability support from extension services, restricted access to improved seed varieties, limited land for food production, and lack of capital for investment.

Livestock owners in arid regions lack the necessary knowledge needed in order to fully understand the implications of drought-tolerant crops in livestock feed systems. For example, in Uganda, it has been reported that the limited adoption of drought-tolerant crops by smallholder farmers included lack of awareness about the crops, which further resulted in low crop production [277]. With respect to the cacti plants, farmers are unaware of the difference between spiny and spineless cactus, leading to the perception that both plants are the same [283]. Meanwhile, in some areas like Pakistan the cultivation of cacti species is not valued, as it is perceived that they are invasive species, rendering their farming fruitless. This perception highlights the lack of awareness, knowledge and training on the use of drought-tolerant crops, for smallholder farmers. The use of drought-tolerant crops for livestock forms part of the climate smart agricultural approach; however, the use of climate smart approaches is hindered by insufficient technical knowledge amongst smallholder farmers [284].

The access to technical information is one of the challenges facing the extended use of drought-tolerant crops in livestock feed. Research results from drought-tolerant crops fail to reach the ultimate beneficiaries. For instance, limited access to improved seed systems for enhanced sorghum varieties by smallholder farmsteads in regions such as Nigeria has been reported [285]. Besides limited access to research information, the available engineered or certified seeds are more expensive compared to the traditional ones [286]. Acevedo et al. [287] reported that both access and lack of information on the certified seed varieties were principal barriers that hindered farmers from adopting climate-resilient crops, especially those with low-income status. These findings underscore a lack of support from agricultural extension services, whose duties include providing support, guidance and information to local farmers.

Another significant barrier to using drought-tolerant crops for livestock feed is the limited land for food production. This challenge is further intensified by water scarcity, land degradation and arability, which limit the resources available for sustainable feed crop cultivation. For instance, South African arable land is only about 12% of the total surface area while the rest is considered for grazing [288]. Unfavorable edaphoclimatic variations are reported to contribute to the observed limited land use for crop production, allowing the irrigation of rain-fed crops only. Lack of capital and monetary support from financial institutions is also hindering the adoption of climate-smart agriculture practices, especially for small-to-medium-scale farmers. Olabanji et al. [289] reported that smallholder farmers in South Africa often face challenges in accessing credit from financial organizations, limiting their participation in climate-smart agriculture interventions. Addressing these issues requires increased investment and support systems to encourage the use of these crops, which further ensure food security and sustainable agricultural practices.

Besides the highlighted socio-economic and financial challenges, farmers, especially commercial farmers, often hesitate to adopt drought-tolerant crops, preferring conventional animal feed due to a complex interplay of economic risks and operational efficiency concerns. These barriers are further compounded by inadequate supply chain and market infrastructure, psychological and behavioral factors, technical and environmental constraints, and broader institutional limitations. Consequently, increasing adoption of drought-tolerant crops remains a significant challenge that requires a holistic solution.

## 7. Key Strategies for Sustainable Innovation for Drought-Tolerant Livestock Feed

### 7.1. Increased Awareness

To effectively address the challenge of limited awareness regarding the adoption of drought-tolerant crops, relevant organizations and institutions (across private, non-governmental, and government sectors) need to establish strong relationships with smallholder farmers in rural settlements. Awareness campaigns and outreach programs, especially in rural farmsteads, are important to strengthen and yield increased adoption of drought-tolerant crops in arid environments. The outreach programs should focus on informing the farmers about available agricultural extension services, knowledge on climate-smart technology, where and how to access drought-tolerant certified seeds, the latest research findings, financial support, and the importance of these crops as livestock feed [286]. Partnerships with key stakeholders such as telecommunication companies, government, and non-governmental organizations will help facilitate the awareness and widespread adoption of drought-tolerant plants in livestock [286]. Zougmoré et al. [290] emphasized the need to increase awareness of climate-smart technologies, especially for vulnerable communities. In some parts of Southern Africa (Zimbabwe and South Africa), there is evident lack of awareness of drought-tolerant crops as a climate-smart agriculture for improved crop production. The adoption of drought-tolerant maize varieties by smallholder farmers has shown increased crop yield compared to those who did not cultivate these varieties [289,290]. Furthermore, their incorporation resulted in increased profits of USD240/ha with no additional cost incurred. This is proof that continuous awareness programs with a specific focus on the adoption of drought-tolerant crops for livestock feed hold a potential that can help improve the performance of livestock in arid regions. This approach will help to disseminate information about climate-resilient plants and their implications in livestock productivity to help small-scale farmers to make informed decisions. This is equally important for established farms whose livestock depends on conventional feed sources and intend on reducing operational costs while maintaining efficient production.

### 7.2. Policy Reforms

Policies supporting innovation of drought-tolerant crops for livestock feed are necessary vehicles that will allow small-scale farmers to access the needed financial support, research and development, and regulatory frameworks. In addition, reforming the existing policies is further needed to ensure inclusion and widespread adoption of drought-tolerant crops as climate-smart agriculture in smallholder farming operations, especially livestock producers. The current policies are reported to be unfavorable for small-scale livestock producers, especially in African countries [291]. Acevedo et al. [287] purported that the existing seed policies have created challenges in accessing the improved seed varieties by the majority of farmers. Wongnaa [291] stresses that for livestock farms to be sustainable during unpredictable climatic events, policy reforms must be prioritized. Nonetheless, there are existing examples of countries that have developed policy frameworks coupled with financial support, implementation and results. Ghana and Nigeria have successfully developed policies planned at implementing climate-smart agricultural practices for farmers vulnerable to drought [292,293]. In Malawi, there is a Climate-Smart Agriculture (CSA) policy that promotes the adoption of drought-tolerant crops, including cassava, sweet potatoes, Irish potatoes, sorghum and millet [294]. To support these initiatives, the International Potato Center provides farmers with improved potato varieties following droughts and during dry seasons. Additionally, the Malawian government encourages practices such as legume cultivation and the intercropping of shrubs with maize to enhance soil quality and crop yields amidst increasing climate variability.

In Mali, policy reforms have enabled the implementation and provision of eco-friendly solutions, such as bio-pesticides, organic fertilizers, fallow and zero tillage, as part of climate change adaptation strategies [291]. In South Africa, the Water Research Commission, a South Africa government entity, and non-governmental organizations such as The Maize Trust, and USAID fund projects that promote intercropping conventional crops with drought-tolerant varieties. These initiatives aim to enhance fodder yield and quality during dry periods, with implementation supported by workshops and on-farm experimentation [295]. In other parts of South Africa, such as Polokwane, Drought Fodder Relief Schemes implemented by Department of Agriculture, Forestry and Fisheries have provided supply fodder to low-resourced farmers during dry seasons [296]; however, further adjustments are recommended to foster the long-term adoption of drought-tolerant crops in these regions. Countries such as Djibouti, Eritrea, Ethiopia, Kenya, Somalia, South Sudan, Sudan, and Uganda, that are part of the Intergovernmental Authority on Development, have developed a regional specific strategy, in the midst of climate uncertainty, to absorb actors from the commercial agricultural value chain to facilitate the promotion and production of drought-tolerant crops and index-based insurance for livestock farmers in those regions. The FAO, in collaboration with the Canadian government, introduced a project that financially supports smallholder farmers to afford certified seeds of drought-tolerant fodder, livestock-breeding tools and equipment [287]. In addition, government investment in research on innovation of drought-tolerant crops in livestock feed is fundamental to developing new, higher-yielding, and nutritious varieties suitable for local conditions. This includes utilizing advanced techniques such as molecular breeding and gene-editing tools. Public–private partnerships, such as the Drought Tolerant Maize for Africa program, an initiative led by International Maize and Wheat Improvement Center and the International Institute of Tropical Agriculture, with an aim to develop and distribute drought-tolerant maize varieties in sub-Saharan Africa is one good example of a successful model for future initiatives.

World Bank investments and loans aimed at reducing livestock losses and fostering climate-resilient farming demonstrate a global shift toward sustainable food systems. Notably, a recent 200 million USD initiative in China supports the implementation of policies that promote drought-tolerant fodder production, aiming to increase livestock productivity while reducing greenhouse gas emissions [297]. Economic forecasts suggest this investment will boost farm profits by 13–19%, depending on farm size and production type (milk, beef, or mutton) [297]. Similar projects financed by the World Bank in Kenya, Costa Rica, Afghanistan, Ethiopia, and India highlight a broader, cross-country adoption of drought-tolerant crops and institutional support [298,299,300,301,302]. These strategic financial interventions and policies underscore the urgent need to integrate drought-tolerant crops as novel feed ingredients in livestock farming, particularly in developing nations where research must focus on evidence-based, rapid adoption [297,298,299,300,301,302]. These initiatives demonstrate a strong, collaborative effort between governments and non-governmental institutions, both national and global, to promote drought-tolerant crops for animal feeding in response to climate change.

### 7.3. Strengthening of Research Capacity

Strengthened research, integrating traditional breeding with cutting-edge biotechnology such as marker-assisted selection and gene editing, is vital to accelerate the development and use of superior drought-tolerant varieties. Research on Sudan sorghum has proven that genetic manipulation, through traditional breeding techniques, is able to produce varieties with optimized growth abilities as it can be propagated in various soil types without the loss of its nutritional value. Research on cultivar breeding of forages is an ideal approach to improve plant resistance, durability and nutritional value of the crops of interest [303]; *Lolium*, *Trifolium* and *Medicago* spp. are crops that have shown greatest improvements through breeding technology [303]. The continuous research on these species has attracted the participation of various institutes across Canada and the United States, showcasing how joint cooperative efforts improve the quality of research findings that benefit research, industries and communities. Similar plant breeding projects exist in countries such as Uganda, Tanzania and Mozambique which aim to explore ways to improve crop varieties to withstand drought intensities [304]. Another example is the collaboration between Mexican, Nigerian, and other Sub-Saharan institutions, which has successfully developed drought-tolerant maize varieties, bringing significant benefits to smallholder farmers.

Despite advancements in research on drought-tolerant crops, a significant disconnect persists among academia, industry, and government, hindering support for research into these crops as alternative feed sources during droughts. As result, research on drought-tolerant crops is not being translated into industrial applications. This gap is particularly evident in South Africa, where research on alternative feed and superior forage options is not only limited [5], but public evidence of collaboration between the government and the private sector is lacking. Although industry and government maintain active breeding programs, academic involvement is less common. Consequently, research institutions contribute minimally to generating the data needed to encourage the wider adoption of drought-tolerant crops among smallholder farmers. By increasing governmental funding for academic research, scientists can create the essential tools needed to help small farmsteads transition to using drought-tolerant feed crops. Thus, collaboration among researchers, government, industry, and farmers is an effective way to optimize the use of these crops for animal feed, which is becoming increasingly important due to unpredictable climate change. Furthermore, private seed and plant-breeding companies can expand their research platforms to include small-scale farmers as active collaborators in developing drought-tolerant crops for feed, rather than focusing solely on commercial operations. By transitioning from centralized models to participatory plant breeding programs, these companies can leverage local knowledge to develop climate-resilient varieties tailored to marginal environments. The empirical results from this inclusive approach provide a vital foundation for evidence-based policy, driving the integration of drought-tolerant crops and advanced processing technologies within the small-to-medium-scale farming sector. Importantly, research investments focused on enhancing the quality, processing efficiency, shelf life, and overall quantity of alternative feed sources (drought-tolerant crops) will boost their adoption by small-scale farmers.

## 8. Conclusions and Prospects

This review critically discussed the role of key drought-tolerant crops—including *S. bicolor*, *P. glaucum*, *E. coracana*, *M. esculenta*, *E. ventricosum*, and *O. ficus-indica*—as alternative livestock feed. It highlighted the main drivers for the use of drought-tolerant crops and provided an in-depth discussion on their processing and application, factors influencing their quality, challenges, and strategies to promote their innovation as climate-smart agriculture strategy. The impact of climate change is the main driver for the application and research of drought-tolerant crops for livestock feed, as climate unpredictability continues to threaten traditional feed resources and livestock productivity. Various techniques such as drying, ensiling, milling, and pelleting have been applied to process drought-tolerant crops. Out of the studies processing techniques, sun drying is still the predominant method for drying drought-tolerant crops, and there is a noticeable absence of advanced drying techniques, indicating a need for research and innovation in this field. The incorporation of alternative feedstuffs such as *S. bicolor*, *P. glaucum*, *E. coracana*, *M. esculenta*, *E. ventricosum*, and *O. ficus-indica* into livestock diets to partially or wholly replace conventional feed has shown promising results in enhancing animal health and nutrition. However, optimizing these formulations using advanced AI techniques such as prediction models could represent a significant research frontier with the potential to produce better results with less use of laborious and destructive methods. Machine learning has been proven in other research spheres to predict outcomes such as plant yield, disease incidence, drought stress and optimum growth. Thus, similar approaches can be integrated into drought tolerant feed formulation systems by manipulating data variables into a machine learning algorithm in order to have feasible and desired outcomes. Also, the formulation of total mixed rations using drought-tolerant crops presents an opportunity that could yield nutritional, livestock performance and economic benefits in small scale and commercial farms. Cultivar and maturity stage are the main preharvest factors affecting the quality of drought-tolerant crops and their livestock feed, whilst after harvesting, drying, grinding and pulverization, ensiling and storage processes are the key determinants of quality preservation and enhancement. There is a need for further research to comprehensively study these crucial quality determinants, especially how they interact with each other and across a diverse range of drought-tolerant crops.

The development of drought-tolerant crops faces several key challenges, including susceptibility to disease and mycotoxins, low protein levels, and issues with digestibility due to high lignin content. Socio-economic and financial obstacles also limit their effectiveness. To fully leverage the potential of these crops for food and nutrition security and climate-smart agriculture, a comprehensive, integrated strategy is necessary to address all these issues. While progress is being made in developing drought-tolerant crops, several key research areas remain under-explored. There is a need to expand research on various drought-tolerant crops as the current research focuses on a limited number of crops mainly, *S. bicolor*, and *P. glaucum* which are crops for human food. Though research has expanded into other crops like *O. ficus-indica* species, this particular crop’s vast potential as a solution to livestock feed shortages in drought-prone areas remains entirely underutilized. Current research on *O. ficus-indica* has primarily focused on the nutritional profiling of the whole cladodes, despite the plant’s overall importance and potential of fruit waste. This approach overlooks the potential for significant nutritional and techno-functional differences in specific components of cladodes like mucilage and fiber, which could greatly influence the properties of feed derived from cladodes. Additionally, studies lack diversity in investigating the influence of cultivar and maturity stage. Although the *O. ficus-indica* is globally distributed, research is geographically sparse. Addressing these critical factors is essential for driving innovation related to this plant. While advanced drying methods such as freeze-drying are not commonly used, they remain critical as the gold standard for preservation. This benchmark is essential for assessing the impact of other processing techniques and underscores the necessity of advanced technology in developing drought-tolerant crops for feed.

The short-term future research strategies for innovating drought-tolerant crops for livestock feed should focus on (1.) identifying more drought-resilient species with drought tolerance, optimizing agronomic and water-management practices, as well as intensifying research on preharvest and processing factors, (2.) application of inorganic and organic nutrients and chemicals to support their growth or enhance the drought tolerance and antioxidant defense systems, (3.) enhancing the microbial fermentation and total mixed rations to enhance the nutritional quality of drought-tolerant crops, while accelerating research into cost-effective strategies for reducing lignin and increasing digestibility, (4.) policy and financial support, (5.) creating integrated research platforms, (6.) creating more awareness on the potential of the drought-tolerant crops as sustainable livestock feed, (7.) carrying out techno-economic and environmental impact assessments of producing drought-tolerant crops and processing them into feed. Meanwhile, the long-term future research strategies should focus on (1.) advanced plant breeding and genetic engineering to develop high-protein drought-tolerant varieties, (2.) exploring novel drought-tolerant crops, (3.) intensifying research on preferred cultivars of drought-tolerant crops for animal feed.

## Figures and Tables

**Table 1:** Proximate composition (% DM) and metabolizable energy (MJ/kg DM) of drought-tolerant crops in comparison with selected commercial feed crops.

	Chemical Component	Fat	CF	CP	Ash	Carbohydrate	ME	References
Drought-tolerant crop	Sorghum	3–5	1–3	6–9	1–2	72–78	14.31–16.35	[53,54]
	Pearl Millet	5–10	2–18	4–17	1–2	55–81	8.4–10.3	[55,56]
	Finger millet	1–2	0.9–10	2.6–14	2–4	68–79	6.5–6.9	[55,57]
	False Banana	0.1–0.6	0.1–0.2	0.6–2	1–2	94–98	4.2–12.2	[58,59]
	* Cassava	0.2–0.6	-	1–2	1–2	89–93	14.2–19.3	[60,61]
	* Cactus	1–2	18–22	4–6	3–7	-	8.8–9.9	[62,63]
Commercial feed crop	Soybean meal	1–2	3–4	46–49	6–7	27–30	9–9.4	[64]
	Maize	1–3	0.9–2	6–8	1–2	79–83	8.7–9.6	[65,66]

Note: Values are reported from different studies. * Cactus units initially reported as g/100 g DW were converted and reported as percentage (%) for uniformity. Cassava and Soybean meal units for ME initially reported as kcal/kg, while for Cactus it was Mcal/kg DM, were converted and reported as MJ/kg DM for uniformity. Abbreviations CF = crude fiber, CP = crude protein, ME = Metabolizable Energy.

**Table 3:** Carbon footprint, water-use efficiency and cost effectiveness of commercial crops and drought-tolerant crops based on different studies.

Metric	Drought-Tolerant Crops (e.g., Cactus, Sorghum, Millet, Cassava)	Commercial Crops (e.g., Maize, Wheat, Alfalfa, Rice Straw, Sugarcane)	References
Carbon footprint	Low (less methane emissions, maintains soil carbon content, lower milk and meat carbon footprint approximately: 1.30 kg CO_2_ eq/kg FPCM and 10.2 kg CO_2_ eq/kg LWG).	High (increased methane emissions, reduced soil carbon content, an estimated equivalent of 6094 kg CO_2_ eq/ha and 2882 kg CO_2_ eq/ha in greenhouse gas emission; 263 kg CO_2_ eq emissions, 685.66 kg CO_2_ eq/t feed).	[150,151,152,153,154]
Water-use efficiency	High (less than 500 L/kg of grain; equivalent to 5.93 kg m^−2^ low evapotranspiration daily rate; high increase of about 30% in water-use efficiency; transpiration ratio of 25–80; low water footprint; reduced crop water use).	Low (equivalent to 5.01 kg m^−2^, high daily rate of evapotranspiration, reduction of about 43% WUE, transpiration ratio of 200–800 and 100–400, high water footprint, high water consumption).	[153,155,156,157,158,159]
Yield	High (fresh forage yield highest leaf area, ear panicle and length growth rates of approximately 0.44 Mg/ha; high dry matter yield).	Low (ear panicle length less than 17.2 cm, growth rate lower than 0.35 Mg/ha [160]; low dry matter yield).	[157,158,159,160]
Input cost	Low (low total variable costs of about 20%; cactus diet lowers feeding costs; minimum land required to produce maximum economic returns; reduction in feed costs).	High (moderately high costs of variables; higher feeding costs; large land size to produce maximum profit margins; high production cost of feed; higher production cost).	[145,154,161,162,163,164]
Economic returns	High (maximum economic gain of approximately 711.6 USD/ha with low inputs, benefit–cost ratio (5.9), high gross margins, internal rate of return equating to about 46%, and high profit margins during drought)	Low (minimum gross margins; lower profitability rate).	[145,158,162,163,164]

Abbreviations: FPCM = Fat and Protein Corrected Milk, LWG = Liveweight gain, WUE = Water Use Efficiency.

## Data Availability

No new data was generated in this review article.

## Abbreviations

3-PGA	3-Phosphoglyceric acid
AI	Artificial intelligence
ADF	Acid detergent fiber
ADG	Average daily gain
ADI	Average daily intake
ADL	Acid detergent lignin
ADP	Adenosine diphosphate
ATP	Adenosine triphosphate
AWG	Average weight gain
BMR	Brown midrib
CAGR	Compound annual growth rate
CAM	Crassulacean acid metabolism
CFU	Colony forming unit
CF	Crude fiber
CNN	Convolutional neural network
CP	Crude protein
CSA	Climate smart agriculture
DM	Dry matter
DMY	Dry matter yield
DPPH	2,2-diphenyl-1-picrylhydrazyl
FAD	Flavin-adenine dinucleotide
FADH_2_	Dihydroflavine adenine dinucleotide
FAO	Food and agricultural organization
FCR	Feed conversion ratio
FPCM	Fat and protein corrected milk
G3P	Glyceraldehyde-3-phosphate
GAE	Gallic acid equivalent
GE	Gross energy
IVDMD	In vitro dry matter digestibility
IVOMD	In vitro organic matter digestibility
LAB	Lactic acid bacteria
LWG	Liveweight gain
ME	Metabolizable energy
MRSA	Methicillin resistant *Staphylococcus aureus*
NAD	Nicotinamide adenine dinucleotide
NADH	Nicotinamide adenine dinucleotide plus hydrogen
NADP^+^	Nicotinamide adenine dinucleotide phosphate (oxidized)
NADP	Nicotinamide adenine dinucleotide diphosphate
NADPH	Nicotinamide adenine dinucleotide phosphate (reduced)
NDF	Neutral detergent fiber
NFE	Nitrogen free extract
OAA	Oxaloacetic acid
OM	Organic matter
ORAC	Oxygen radical absorbance capacity
PEP	Phosphoenol pyruvate
RGB	Red green blue
RFV	Relative feed value
RuBp	Ribulose-1,5-bisphosphate
SDDGs	Soluble dried distiller’s grains
SFM	Sunflower meal
SW	Sweet sorghum
WSC	Water soluble carbohydrates
WUE	Water use efficiency
